# Colon cancer molecular subtypes identified by expression profiling and associated to stroma, mucinous type and different clinical behavior

**DOI:** 10.1186/1471-2407-12-260

**Published:** 2012-06-19

**Authors:** Beatriz Perez Villamil, Alejandro Romera Lopez, Susana Hernandez Prieto, Guillermo Lopez Campos, Antonio Calles, Jose Antonio Lopez Asenjo, Julian Sanz Ortega, Cristina Fernandez Perez, Javier Sastre, Rosario Alfonso, Trinidad Caldes, Fernando Martin Sanchez, Eduardo Diaz Rubio

**Affiliations:** 1 Molecular Oncology Laboratory, Medical Oncology Department, Hospital Clinico San Carlos, Instituto de Investigacion Sanitaria del Hospital Clinico San Carlos (IdISSC), C/ Martin Lagos s/n, Madrid, 28040, Spain; 2 Department of Surgical Pathology, Hospital Clinico San Carlos, Instituto de Investigacion Sanitaria del Hospital Clinico San Carlos (IdISSC), C/ Martin Lagos s/n, Madrid, 28040, Spain; 3 Medical Bioinformatics Department, National Institute of Health Carlos III, Ctra, Majadahonda a Pozuel, Km. 2 Majadahonda, 28220, Madrid, Spain; 4 Department of Preventive Medicine, Hospital Clinico San Carlos, Instituto de Investigacion Sanitaria del Hospital Clinico San Carlos (IdISSC), C/ Martin Lagos s/n, Madrid, 28040, Spain; 5 Health Informatics. Melbourne Medical School, Faculty of Medicine, Dentistry & Health Sciences. Health and Biomedical Informatics Research (HBIR) Unit IBES-Institute for the Broadband-Enabled Society, The University of Melbourne, Level 1, 202 Berkeley Street, 3010, Melbourne, VIC, Australia

**Keywords:** Colon cancer, Microarray gene expression, Molecular classification, Stroma, Survival

## Abstract

**Background:**

Colon cancer patients with the same stage show diverse clinical behavior due to tumor heterogeneity. We aimed to discover distinct classes of tumors based on microarray expression patterns, to analyze whether the molecular classification correlated with the histopathological stages or other clinical parameters and to study differences in the survival.

**Methods:**

Hierarchical clustering was performed for class discovery in 88 colon tumors (stages I to IV). Pathways analysis and correlations between clinical parameters and our classification were analyzed. Tumor subtypes were validated using an external set of 78 patients. A 167 gene signature associated to the main subtype was generated using the 3-Nearest-Neighbor method. Coincidences with other prognostic predictors were assesed.

**Results:**

Hierarchical clustering identified four robust tumor subtypes with biologically and clinically distinct behavior. Stromal components (p < 0.001), nuclear β-catenin (p = 0.021), mucinous histology (p = 0.001), microsatellite-instability (p = 0.039) and BRAF mutations (p < 0.001) were associated to this classification but it was independent of Dukes stages (p = 0.646). Molecular subtypes were established from stage I. High-stroma-subtype showed increased levels of genes and altered pathways distinctive of tumour-associated-stroma and components of the extracellular matrix in contrast to Low-stroma-subtype. Mucinous-subtype was reflected by the increased expression of trefoil factors and mucins as well as by a higher proportion of MSI and *BRAF* mutations. Tumor subtypes were validated using an external set of 78 patients. A 167 gene signature associated to the Low-stroma-subtype distinguished low risk patients from high risk patients in the external cohort (Dukes B and C:HR = 8.56(2.53-29.01); Dukes B,C and D:HR = 1.87(1.07-3.25)). Eight different reported survival gene signatures segregated our tumors into two groups the Low-stroma-subtype and the other tumor subtypes.

**Conclusions:**

We have identified novel molecular subtypes in colon cancer with distinct biological and clinical behavior that are established from the initiation of the tumor. Tumor microenvironment is important for the classification and for the malignant power of the tumor. Differential gene sets and biological pathways characterize each tumor subtype reflecting underlying mechanisms of carcinogenesis that may be used for the selection of targeted therapeutic procedures. This classification may contribute to an improvement in the management of the patients with CRC and to a more comprehensive prognosis.

## Background

Colorectal cancer is one of the most common malignancies in the western world and accounts for about 10% of all cancer deaths in both Europe and the USA. Traditionally, colorectal cancer classification (Dukes, AJCC (American Joint Committee on Cancer)) is based in the extent of the cancer: depth of tumor invasion into the wall of the intestine, number of nearby affected lymph nodes and whether the cancer has metastasized to other organs of the body. Surgery is curative for a big proportion of patients at early stages, but is not enough for many patients at advanced stages. Most of these patients need adjuvant chemotherapy in order to avoid relapse or to increase survival. Unfortunately, only a small portion of them shows an objective response to chemotherapy, becoming problematic to correctly predict patients’ clinical outcome [1]. Microarray gene expression profiling is a powerful tool for the identification of prognostic gene signatures. Supervised analysis of gene expression has been used to discover gene signatures to identify patients at risk of recurrence in colon cancer [2-8]. Recently two extensively validated gene signatures have been reported Oncotype-DX and ColoPrint [9,10]. A different approach is to use unsupervised analysis. Clustering methods group together samples with similar expression profiles. With this strategy, new subtypes of tumors can emerge or the existing classification may be redefined with the result of more uniform groups of tumors. Molecular homogeneity may be essential in order to identify specific biological pathways affected, to discover precise drug targets in each subgroup or to obtain individual survival classifiers. Previous attempts to subdivide colon tumors into sub-classes or to correlate gene expression to Dukes stages using unsupervised analysis haven’t been conclusive. Some authors were able to correctly classify normal colon, Dukes B and C but not Dukes A and D and no new subgroups were identified [11]. Others were able to classify in one group normal tissue with Dukes A, in another cluster B with C, and D clustered separately [12]. Other authors were unable to find differences between stages B, C and D [13]. Some reports found differences between normal and tumor tissue and genes differentially expressed between metastatic and nonmetastatic samples [14-16] or segregate normal tissue from primary carcinomas and from liver metastasis and carcinomatoses [14,17]. Other authors using class comparison between Dukes A and D identified a gene signature that could be used for the classification of low- and high-risk patients in Dukes B and C [7]. Another interesting approach described the identification of a gene expression profile generated from an experimental model of colon cancer metastasis that was able to predict cancer recurrence in patients with colon cancer [18]. Other authors reported that epidermal growth factor receptor pathway was up-regulated in metachronous liver metastasis while angiogenesis was up-regulated in synchronous liver metastasis [19]. Unfortunately, even if there is an ample selection of gene signatures reported in the literature, almost none of them have reached the clinical practice. There is a need of prognostic and predictive factors to provide authoritative information for medical decisions in routine clinical practice. Our study was mainly aimed to obtain more homogeneous groups of tumors in colorectal adenocarcinomas hypothesizing that discovering molecularly more uniform groups of tumors, would likely discriminate patients with different clinical outcomes, as well. In addition understanding the biological pathways underlying each tumor subtype would potentially help in future, to find the appropriate treatment regime.

## Methods

### Patients

Patients from all stages were selected, keeping approximately equal proportion of each stage (24 Dukes A or AJCC (6 edition) stage I; 26 B or II; 19 C or III and 19 D or IV)). Tumor samples were taken from the Bank of Tumors of the Hospital Clinico San Carlos between 2001 and 2006. The Bank of Tumors follows the rules established by the hospital including the patient consent approved by the Ethical Committee of the Hospital Clinico San Carlos.

### Histological analysis of tumor samples

Many reports have shown the importance of tumor associated stroma in the development of cancer; therefore for our study we did not consider to do laser microdissection to get just the transformed epithelial cells. We wanted to analyze both, tumor cells from the malignant epithelia and the altered surrounding stroma. We took a representative fragment of the complete tumor and we carried out a very detailed pathological analysis of the frozen tumor fragments used to extract the RNA and of the corresponding paraffins of the tumor. Only samples with more than 80% of tumor component were included, considering tumor stroma as part of the tumor component.

### RNA extraction and quality control

RNA was extracted directly from the frozen samples using TRIZOL (Invitrogen, Carlsbad, CA) and a homogenizer (Ultraturrax T8-S8N-5 G Rose Scientific Ltd, Canada). Afterwards, RNA was treated with DNAse using RNeasy Microkit (Qiagen GmbH, Germany). RNA quality was measured with Agilent Bioanalyzer 2100 (Agilent technologies, Palo Alto U.S.A) and only good quality samples, RIN (RNA Integrity Number) [20,21] higher than 7.5, were selected for the analysis.

### Microarray analysis

Agilent G4112 microarrays were used to analyze gene expression in 88 colon tumors and 7 normal colon tissues. A reference RNA preparation (pool of normal colon tissue RNAs obtained from 68 individuals) was used for double hybridization: tumor-Cy5/pool-Cy3, normal-Cy5/pool-Cy3. Agilent recommended protocols were followed. Fluorescence was measured and normalized (LOWESS) using Agilent microarray scanner and Feature Extraction software. Quality Control Report was carried out to discard the microarrays that did not fulfill good quality criteria. From the original 44 K features microarray, a total of 28462 spots without flags in 90% of the microarrays were used. Only probes that were significantly (p < 0.01) up or down regulated *vs.* the reference pool, in at least 7 samples (considering the 7 normal tissue samples as the smallest group) were selected to obtain 17392 spots. Probes with the same gene identification were averaged to obtain a total of 14764 genes. For classification purposes we chose the genes that showed higher variations between tumors, selecting the genes that in more than 7 samples had at least a 2.5-fold change from the gene median value, resulting 1722 genes that were used for the unsupervised analysis of the 89 samples (tumor CT102 was replicated). Cluster reproducibility was measured by the robustness index (R-index) and by the discrepancy index (D-index); [22] analyses were performed using BRB-ArrayTools developed by Dr. Richard Simon and BRB-ArrayTools Development Team. Transcript Profiling: [ArrayExpress E-TABM-723].

### Functional analysis of KEGG pathways

A functional analysis of KEGG pathways using class comparison tools (Goeman’s global, LS, KS Efron. Tibshirani’s tests) was carried out to find differentially affected pathways between the four tumor subtypes. 164 gene sets were studied and the threshold used was set at p = 0.005. Multiple comparisons were corrected using resampling and gene permutations. Since Goeman's method tests the null hypothesis that no genes within a given gene set are differentially expressed and LS test, KS test and Efron-Tibshirani's methods, test the hypothesis whether the average degree of differentially expression is greater than expected from a random sample of genes (BRB-ArrayTools), KEGG pathways selected had to be significant at least in two tests: Goeman’s test and any of the other three tests carried out.

### Tissue microarrays (TMA), IHC and mutation analysis

Tissue microarrays were assembled as in [23] for immunological analysis of β-catenin (clone17c2 Novocastra Laboratories Ltd. Newcastle upon Tyne, UK), M30 (M30 CytoDEATH Roche Diagnostics GmbH Mannheim Germany) for apoptosis and KI67 (clone M1B1, Dako, Glostrup, Denmmark) for proliferation. Presence of mutations in *KRAS*, *BRAF* and *PI3K* as well as microsatellite instability (MSI) were also assessed. See Additional file 1: Supplementary Information for more information about the protocols followed for antibody staining and analysis of MSI and gene mutations.

### Identification of tumor subgroups in an independent data set

Eschrich et al. [2] data set was used as an external patient collection. Data was combined using the method published by Hu et al. [24]. The genes that had the same UniGene Cluster ID were averaged and the genes that did not have a UniGene Cluster ID were eliminated from our data set resulting 11017 genes out of the 14764 genes and 96 samples (normal and tumor samples). Eschrich data set consists of 78 samples (23B, 22 C, 30D and 3 adenomas) and 32208 normalized transcripts. Spots without IDs or with more than 25% missing values were eliminated and spots with the same UniGene Cluster ID were averaged. Genes with 90% of data were selected to obtain a total of 9229 genes. **Combination of data sets**: both data sets were combined using the software “Distance Weighted Discrimination” (https://genome.unc.edu/pubsup/dwd/) to obtain a collection of 174 samples (166 tumors) and 5319 common genes. **Classification of the external data set**: A Nearest Centroid predictor was built in our data set including only genes differentially expressed between classes at a p < 0.001. LOOCV (Leave-One-Out Cross-Validation) and 100 random permutations were used to compute miss-classification rate. This predictor was subsequently used to classify the external samples into the four novel clusters. **Hierarchical clustering**: To analyze whether the external patient’s set clustered with our patients in the same tumor subtypes, Centered Pearson correlation and average-linkage-hierarchical clustering of the combined set (159 tumor samples excluding samples from cluster-5, normal tissues and adenomas) was carried out using the 461 common genes between both data sets out of the 1722 originally selected genes.

### Generation of a low-stroma-subtype predictor

Eschrich samples were classified as belonging to the Low-stroma-subtype or belonging to the other tumor subtypes using the K-nearest-neighbor, K = 3 (KNN3) prediction method. A predictor was generated in our data set using the 461 common genes between both data sets out of the 1722 originally selected genes. Genes included in the predictor were differentially expressed between classes at a p < 0.001. LOOCV and 100 random permutations were used to compute miss-classification rate.

### Statistical analysis and correlations with clinical parameters and survival analysis

Qualitative variables are given with their frequency distribution. Quantitative variables are given with their mean and standard deviation (SD). Means were compared with Kruskal-Wallis test. Proportions were compared by the chi square test for independent groups. Survival functions were estimated by the actuarial method. Cumulative risks over time and their corresponding standard errors (SE) are provided along with the number of patients at risk (n). Likelihood exact test was used to compare survival functions for the different subgroups. A Cox's proportional hazards regression model was fitted. Significance was taken as a drop in the likelihood estimator of the models compared. Adjusted hazard ratios (HR) and its 95% confidence interval (95%CI) are provided in the results. In each hypothesis contrast the assumption of rate proportionality was verified. In all hypothesis contrasts (survival analysis and clinical parameters correlations) the null hypothesis of no difference was rejected with a type I or α-error of less than 0.05. Correction of p-values was not performed. Statistical analysis was performed with SPSS 15.0 for Windows (SPSS Inc., Chicago, USA).

## Results

### Identification of tumor subtypes by hierarchical clustering

**Figure 1:**
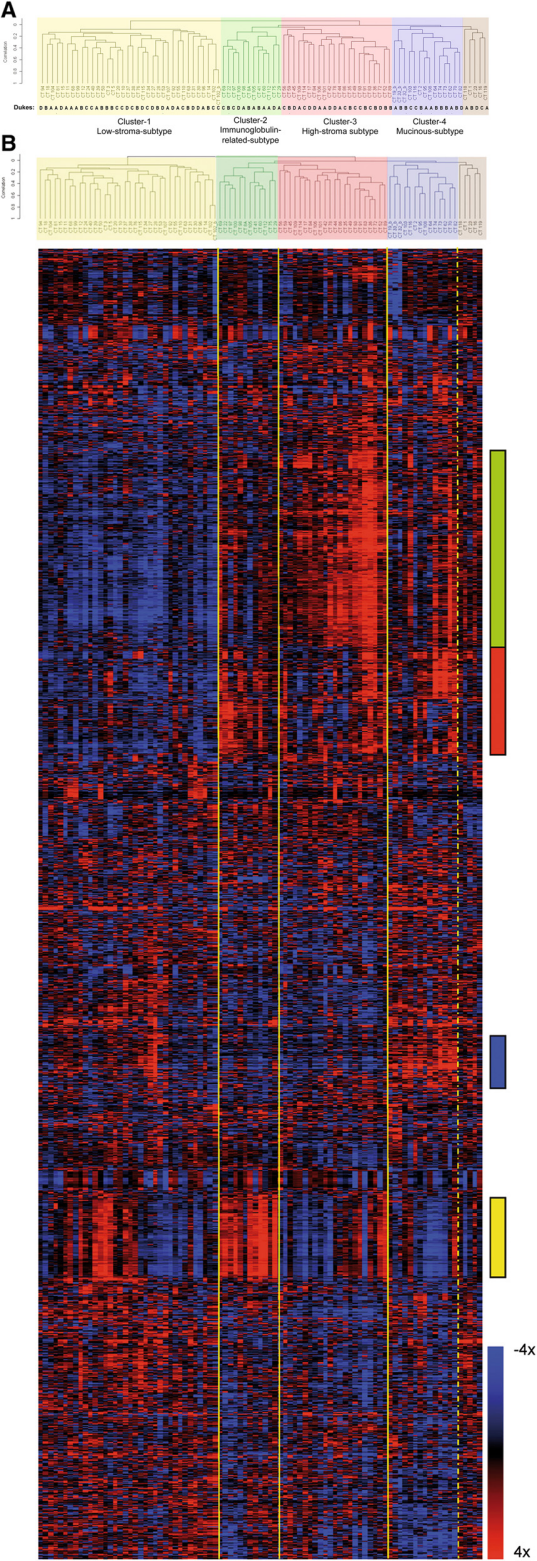
**Hierarchical clustering of the colon tumor samples and the 1722 selected genes A**) Classification of the 89 (sample CT102 is replicated) tumor samples in four main clusters. Yellow shadow: Cluster-1, Low-stroma-subtype; green: Cluster-2, Immunoglobulin-related-subtype; red: Cluster-3, High-stroma-subtype; blue: Cluster-4, Mucinous-subtype; and brown: Cluster-5, unclassified samples. Dukes’ stages are specified below the tree. **B**) Each column in the heatmap represents one sample and each row represents one gene. The relative levels of gene expression within each sample against the reference pool are illustrated according to the color scale bar (4 fold induction (red); -4 fold inhibition (blue)). The principal sections of the graph that distinguish between clusters are indicated with a color bar on the right. The green bar localizes gene 270 to gene 523, a group of 253 genes with low expression in cluster 1 and high expression in cluster 3; the red bar corresponds to genes 524 to 664 a group of 140 genes, whose expression is lower in cluster 1. Blue bar localizes genes 997 to 1102 a group of 105 genes with a higher expression in cluster 4 compared to 3. Yellow bar localizes genes from 1247 to 1352 a group of 105 genes specific for cluster 2 (see Supplemental data for the list of the 1722 genes).

Centered Pearson correlation and average-linkage-hierarchical clustering with the 1722 selected genes was used to group both tumors and genes. The 89 tumor samples (tumor CT102 is duplicated) were arranged primary in two main groups (Figure 1A). The first group contains just one class, cluster-1, with 36 tumors (40% of the total number of samples). The second main group holds the rest of the tumors that were classified in three smaller reproducible subgroups, clusters-2, -3 and −4 containing 12, 22 and 14 tumors respectively (Figure 1A). The robustness and reproducibility of the four new clusters was high, mainly for clusters-1, -3 and −4 with robustness close to 0.9 and a low number of samples additions or omissions. Cluster-2 was the weakest of the four clusters with a robustness of 0.75 (Table 1). Hierarchical clustering identified a fifth cluster with five elements in it and a lower robustness; we did not regard this 5th cluster as a group considering those samples as unclassified tumors.

**Table 1:** Cluster-specific reproducibility

**Cluster**	**Samples (n)**	**Robustness**	**Omissions**	**Additions**
**Cluster 1**	36	0.90	2.54	1.78
**Cluster 2**	12	0.75	2.24	4.98
**Cluster 3**	22	0.87	1.78	2.34
**Cluster 4**	14	0.89	0.92	0.58
**Cluster 5**	5	0.65	1.29	1.56

1000 perturbations were used; SD = 0.439. Overall reproducibility: R-Index = 0.88; D Index = 10.004.

The dendrogram of the 89 tumor samples and the 1722 selected genes is shown in Figure 1B. The principal sections of the graph that distinguish between clusters are indicated with a color bar on the right (Additional file 2: List of 1722 genes see supplementary material). The green bar corresponds to a group of genes with lowest expression in cluster-1 and highest expression in cluster-3 and is enriched in genes that have been reported to be specifically activated in tumor-associated stroma and molecules implicated in extracellular matrix remodeling and cell migration. Since these two clusters were mainly characterized by differences in the abundance of stromal genes, cluster-1 and cluster-3 were defined as the Low-stroma-subtype and the High-stroma-subtype respectively. The red bar localizes genes with lower levels in cluster-1 but their expression is not higher in cluster-3 than in clusters-2 and −4. Distinctive genes of this section are metallothioneins, metallopeptidases and SPP1. Blue bar show the location of genes up-regulated in Cluster-4. These are molecules typically associated to the mucinous type of adenocarcinomas like trefoil factors and mucins, for these characteristics cluster-4 was defined as the Mucinous-subtype. Yellow bar localizes the genes up-regulated in cluster-2. This cluster is mainly characterized by a collection of immunoglobulin-related molecules, for this reason cluster-2 was named as the Immunoglobulin-related-subtype.

### Functional analysis of KEGG pathways

KEGG pathways analysis sustains the implication of the tumor microenvironment in the identified tumor subgroups (see Additional file 1: Table S1 for the list of deregulated KEGG pathways). Pathways corresponding to cell communication, ECM-receptor interaction, Focal adhesion and Cell adhesion molecules showed differences between clusters; elements of these pathways showed significantly lower values in the Low-stroma-subtype than in the other tumor subtypes. Other significant deregulated pathways showing differences among clusters were WNT and TGF pathways.

### Correlations of tumor subtypes with clinical parameters

**Figure 2:**
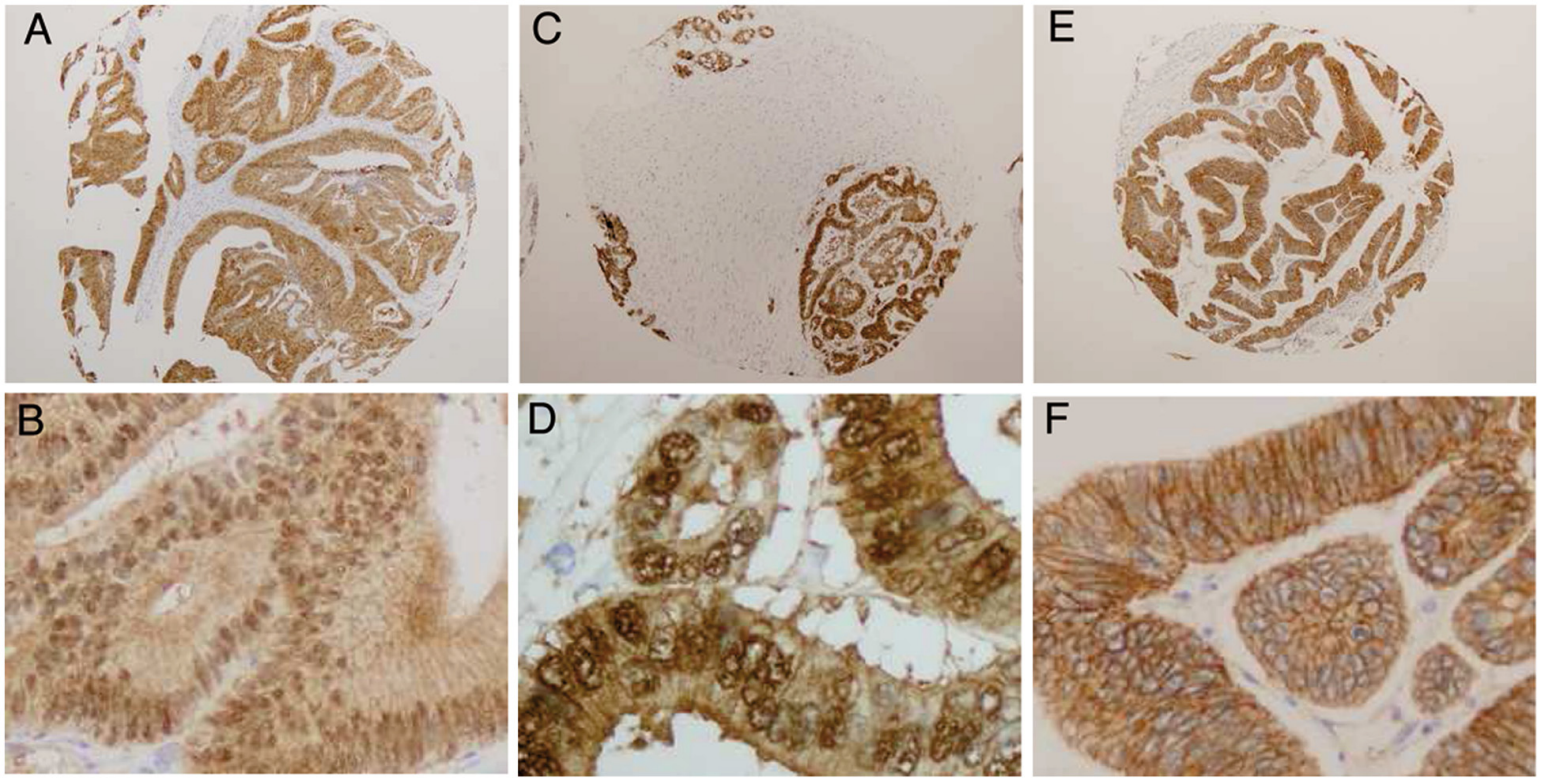
**Immunohistochemistry with β-catenin antibody of tissue microarrays 200X amplification A, C and E; 400X amplification B,D and F.** Sample from tumor CT5 corresponding to Low-stroma-subtype (**A**,**B**); Sample from tumor CT42 corresponding to High-stroma-subtype (**C**,**D**); sample from tumor CT103 corresponding to Mucinous-subtype (**E**,**F**). Note nuclear staining of β-catenin of CT5 and CT42 in contrast to membrane β-catenin staining of CT103.

The Patients’ characteristics are summarized in Table 2. Correlation analysis was performed to find associations between the four novel clusters and clinical parameters (Table 3 and Additional file 1: Table S2). Dukes stages did not show any association (p = 0.646) with the identified tumor subgroups (Figure 1A, Table 3). Parameters that showed a clear correlation with the identified tumor subtypes were: proportion of stroma in the tumors, mucinous histology, the extent of nuclear β-catenin staining, MSI and the V600E *BRAF* mutation. Proportion of tumor stroma in the frozen fragments and in the corresponding paraffin blocks were compared and no differences were found (Intraclass Correlation-ICC = 0.781 (IC95% (0.681-0.852) p < 0.001). Amount of stroma was always the lowest in cluster-1 and the highest in cluster-3; differences were significant between these two clusters in both, frozen samples (p < 0.001) and paraffins (p = 0.005). Tumors in cluster-2 had similar amount of stroma than tumors in cluster-4. The quantity of stroma was significantly lower in cluster-1 related to cluster-2 (p = 0.02) and cluster-4 (p = 0.013) in the frozen samples. Mucinous histology was correlated significantly with this classification (p = 0.001). Pair wise comparisons showed that there was a significant association of the mucinous histology, the MSI tumors and B-Raf mutations to cluster-4 (Table 3). Nuclear β-catenin was also associated with the clusters (Table 3; Figure 2). Pair wise comparisons showed an increased proportion of epithelial cells with nuclear β-catenin in cluster-1 and −3 and a low proportion in cluster-2 and −4. The presence of mutations in K-Ras (codons 12/13) and PI3K (exons 9/20), proliferation (Ki67), apoptosis (M30) as well as other histological parameters did not show association with the molecular subtypes (see Additional file 1: Table S2 for all parameters studied).

**Table 2:** Patients characteristics

		Patients (n)	Percentage
**Sex**	Men	43	48.9%
	Women	45	51.1%
**Age (years)**	Mean (min-max)	70.9	(25–93)
**RIN**	Mean (min-max)	8.9	(7.5-10)
**Duke's Stage**	A	24	27.27%
	B	26	29.55%
	C	19	21.59%
	D	19	21.59%
**MSS/MSI**	MSS	79	89.8%
	MSI	9	10.2%
**Location**	Ascending C.	33	37.5%
	Sigmoid	32	36.4%
	Rectum	14	15.9%
	Descending C.	3	3.4%
	Transverse C.	6	6.8%
**Mut. PI3K**	Exon 9	12	13.6%
	Exon 20	8	9.1%
	Wild Type	68	77.3%
**Mut. B-Raf**	Mutated	8	9.1%
	Wild Type	80	90.9%
**Mut. K-ras**	Codon 12	28	31.8%
	Codon 13	6	6.8%
	Wild Type	54	61.4%
**Histologic grade**	Undifferentiated	1	1.1%
	Poorly diff.	5	5.7%
	Moderately diff.	51	58.0%
	Well diff.	31	35.2%
**Histologic subtypes**	Conventional	78	88.64%
	Mucinous	10	11.36%
**Tumoral margin**	Expansive	16	18.18%
	Infiltrative	61	69.32%
	Mixed	11	12.50%
**Vascular invasion**	Yes	33	37.50%
	No	55	62.50%
**Perineural invasion**	Yes	8	9.09%
	No	80	90.91%
**Lymphocyte infiltration**	Absent	53	60.23%
	Low	15	17.05%
	Medium	17	19.32%
	High	3	3.41%

RIN: RNA integrity factor.

**Table 3:** Correlation of tumor subgroups with clinical parameters

**Parameter**		**Cluster-1 (n = 35)**	**Cluster-2 (n = 12)**	**Cluster-3 (n = 22)**	**Cluster-4 (n = 14)**	**p-global**
Microsatellite instability	MSS	33	11	21	9	0.039^LR,a^
	MSI	2	1	1	5	
Histologic subtypes	Conventional	34	12	21	8	0.001^LR,b^
	Mucinous	1	0	1	6	
% Stroma content frozen sample	Mean	11.2	18.8	23.4	18.9	<0.001^KW,c^
	SD	6.0	10.3	10.5	14.8	
% Stroma Paraffin	Mean	12.6	17.1	18.9	16.8	0.047^KW,d^
	SD	6.0	9.9	8.6	14.2	
nuclear β-Catenin (IHC)	LOW	21	9	11	12	0.021^LR,e^
	MEDIUM	4	0	6	1	
	HIGH	10	2	5	0	
BRAF mutations (V600E)	Mutated	0	1	1	6	<0.001^LR,f^
	WT	35	11	21	8	
Dukes Stage	A	9	5	4	4	0.646^LR^
	B	9	3	6	7	
	C	8	2	6	2	
	D	9	2	6	1	

### Recognition of tumor subtypes in an external clinical cohort

To classify Eschrich samples into the four novel clusters, a classifier of 1039 genes was generated using the 5319 common genes between both data sets and the Nearest Centroid method (85% correct classification). Afterwards, unsupervised analysis of the combined set of 159 tumor samples was carried out. Hierarchical clustering, using 461 genes, associated Eschrich’s samples classified as Low-stroma subtype with our Low-stroma subtype samples; Eschrich’s samples classified as High-stroma-subtype with our High-stroma-subtype samples and his tumors in the Mucinous-subtype with our samples in Mucinous-subtype. Samples of the immunoglobulin related subtype did not show a good association (see Additional file 3: Figure S1). We also used other prediction methods like K-Nearest-Neighbor, (K = 1 and K = 3) and Diagonal Linear Discriminant, obtaining similar results (not shown).

### Differences in survival time between the identified tumors subtypes

Our set of patients was too heterogeneous to analyze survival since it was mainly aimed to obtaining a comprehensive classification of colon cancer. From the total of 88 patients, 26 did not had at least 36 months of following up and other 23 patients were under different treatment schemes. Among the 39 untreated patients there were just one death and four relapses. Under these circumstances, the number of events was not enough to obtain reliable results in the survival analysis.

### Reported survival predictors identified the patients of the low-stroma-subtype

**Figure 3:**
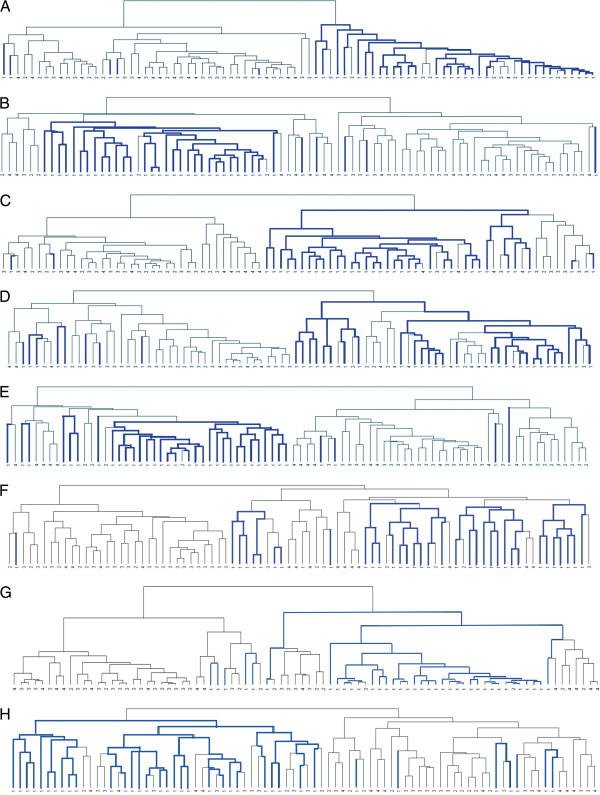
**Hierarchical clustering of our 84 tumor samples A**) using the 17 genes that coincides in both sets out of the total of 43 genes predictor of Eschrich et al.; **B**) using 37 genes out of the 50 genes predictor of Garman et al.; **C**) using 11 of the 23 genes of the predictor of Wang et al.; **D**) using 17 of the 22 genes of the predictor of Lin YH et al.; **E**) using 115 of the 128 genes of the predictor of Jorissen RN et al.; **F**) using 22 of the 34 genes of the predictor of Smith JJ et al.; **G**) using 6 of the 7 genes of the Oncotype-DX predictor; H) using 17 of the 18 genes of the ColoPrint predictor. Blue line: tumors belonging to cluster-1 (Low-stroma-subtype). Black line: tumors from clusters-2, -3 and −4. Note that almost all samples from the Low-stroma-subtype stay together in one group.

To analyze differences in survival of the novel clusters, first we took advantage of the survival predictors already published. We analyzed whether Eschrich et al. [2] 43 genes survival predictor recognized specifically any of our tumor subtypes. Hierarchical clustering of our 84 tumor samples (excluding the samples from cluster-5), using the 17 common genes out of the 43 genes predictor, segregated the samples into two clusters, the first was composed mainly of tumors of the Low-stroma-subtype and the second was composed of tumors of the other subtypes. We also used the predictors of Garman et al. [8] Wang et al. [3]; Lin et al. [4]; Jorissen et al. [7]; Smith et al. [18]; O'Connell et al. (Oncotype-DX) [9] and of Salazar et al. (Coloprint) [10] obtaining similar results (Figure 3). However, other reported predictors such as Barrier et al. [5] and Arango et al. [6] were unable to specifically recognize any of our molecular subtypes (not shown).

### The external patients classified as belonging to the low-stroma-subtype showed better survival

**Figure 4:**
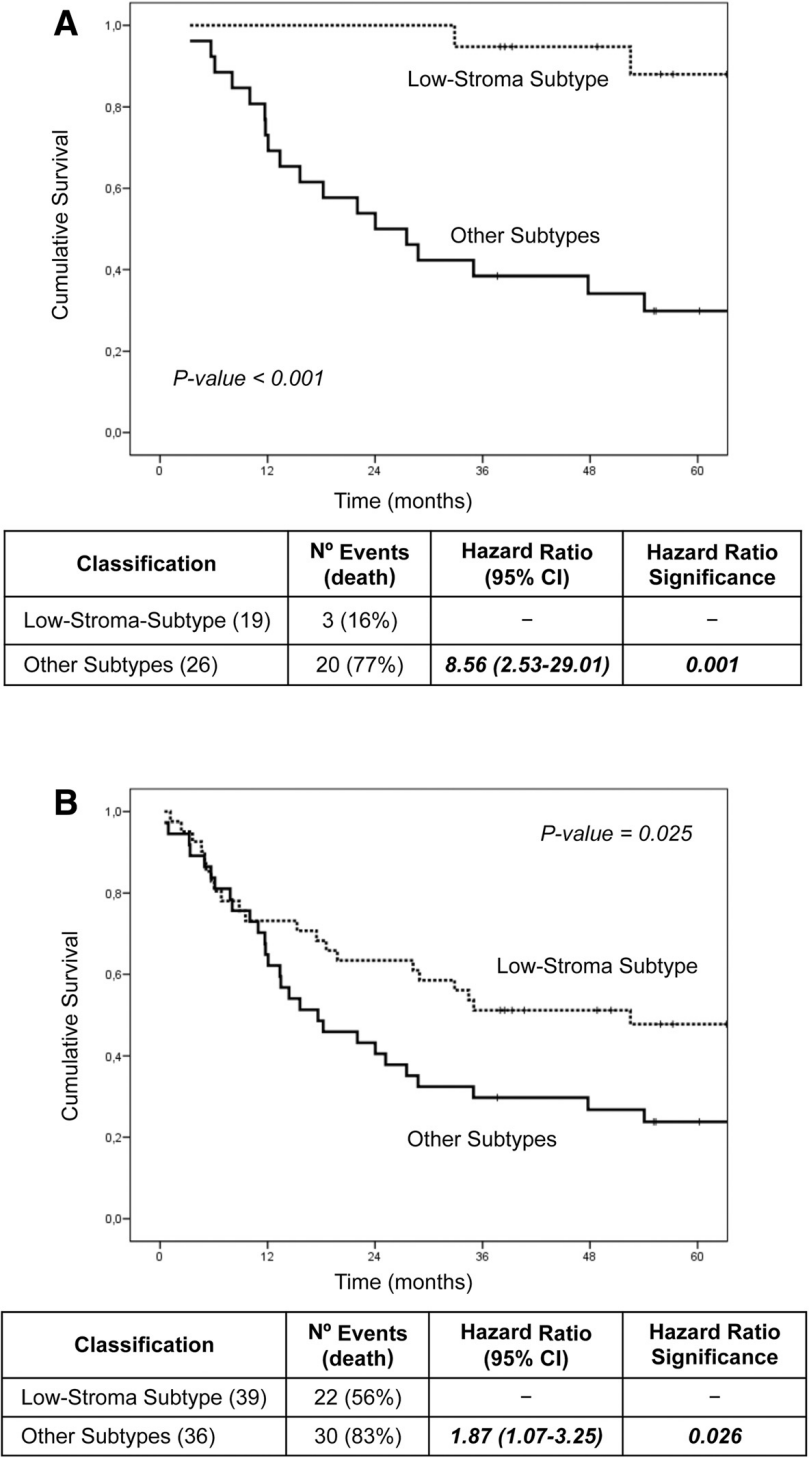
**Kaplan-Meier overall survival analysis of Eschrich patients. A**: (Dukes B and C); **B**: (Dukes B, C and D) classified as belonging to the Low-stroma-subtype or belonging to the other tumor subtypes using the 167 genes Low-stroma-subtype predictor. Number of patients classified in each class, hazard ratio and p values are indicated.

Since Eschrich’s and other published predictors mainly segregated the samples of the Low-stroma-subtype, next step was to address whether our Low-stroma-subtype predictor was able to identify in Eschrich’s data set, the patients with good prognosis. Using the KNN3 classification method and the 461 common genes between both data sets out of the 1722 selected genes, a predictor of 167 genes was generated (see Additional file 4: supplemental information for the list of 167 genes); 96% of correct classification was obtained (see Additional file 1: Table S3 for classification performance). Kaplan-Meier overall survival analysis of Eschrich’s patients classified as belonging to the Low-stroma-subtype showed better survival than the patients belonging to the other tumor subtypes. Low-stroma-subtype patients showed better survival when analyzing both, stages B and C only (Figure 4A) and stages B, C and D (Figure 4B). We also used the Nearest Centroid method finding similar results (not shown).

### Coincidence among predictors

Usually there is a minimal overlap among reported high risk gene signatures [25]. Our Low-stroma-subtype predictor showed some overlapping (12 genes in common) with Jorissen et al. [7] predictor, two genes in common with Eschrich et al. [2] and with Oncotype-DX [9] predictors; one gene in common with the predictors of Garman et al. [8] Wang et al. [3] and ColoPrint [10]. There were no genes in common with Lin et al. [4] and with Smith et al. [18] predictors (see Additional file 1: Table S4 for the list of overlapping genes). Even though there was little or none overlapping, all these eight reported predictors recognized the tumors of the Low-stroma-subtype.

## Discussion

A general approach to find prognostic markers in colon cancer is using supervised analysis of gene expression. Class comparison between patients with good and bad prognosis has been carried out, and gene signatures that discriminate between high and low risk patients have been reported [2-10]. In this study, we have used a different strategy, hypothesizing that the identification of distinct molecular tumor subtypes would likely discriminate patients with different clinical outcomes, as well. In addition understanding the biological pathways underlying each tumor subtype would likely help to find the appropriate treatment scheme.

We report a molecular classification of colon adenocarcinomas in four novel tumor subtypes identified by unsupervised analysis of gene expression. Tumor-associated-stroma was clearly associated with this classification characterizing a Low-stroma-subtype and a High-stroma-subtype. Mucinous histology, MSI, *BRAF* mutations as well as lower levels of nuclear β-catenin characterize the Mucinous-subtype. Tumor subtypes were independent of the histopathological stages. Lack of association with the histopathological staging is important, because it implies that tumor subtypes are established since initial stages of the tumor, consequently contributing to the selection of the patients at early stages. Additionally, explains why many studies were unable to reliably associate molecular classification to Dukes stages [11,12,14,17]. The nature of the genes expressed in each cluster and the biological pathways affected supported the association of the molecular and pathological parameters with the tumor subgroups. Low-stroma-subtype, High-stroma-subtype and Mucinous-subtype were robust, associated to biological characteristics and validated in an external patient set. The combination of two different microarray studies in one data set is challenging; many of the important genes in each data set may be lost in the merged spreadsheet. Even though, when we combined our data set with the external data set we still kept important features in the combined data set. The novel molecular subtypes were also identified in the external data set (at least three of the four clusters).

Relevant reports identified stroma gene signatures associated to survival in diffuse large-B-cell lymphoma [26] and in breast cancer [27] reflecting the importance of tumor microenvironment in the aggressive progression of the disease [28]. Moreover a report in colon cancer showed that the presence of a high amount of stroma, predicts worse survival for stage I-II colon cancer patients [29]. Stroma was highly associated to our molecular classification. Genes corresponding to pathways related to cell communication, ECM-receptor interaction, Focal adhesion and CAMs were down-regulated in the Low-stroma-subtype and up-regulated in the High-stroma-subtype and in the Mucinous-subtype. High-stroma-subtype had the highest percentage of stroma in the tumors and the highest level of stromal components. Mucinous-subtype also had high levels of stroma associated genes and the proportion of stroma was not significantly lower than in the High-stroma-subtype. Although clusters-3 and −4 share similar expression patterns of some of these stromal genes, there are other important genes that clearly are different between these two subtypes, genes characteristic of goblet cells, trefoil factors and mucins, as well as other genes like *REGIV*, *COX2* or *CD55* are specifically up-regulated in cluster-4 or Mucinous-subtype.

Microenvironment is important for tumor development and more interestingly may be the target of novel treatments. In this line, promising studies are underway. Although initial studies using antibodies against activated fibroblast proteins, like *FAP,* did not obtain objective tumor responses [30]. New developments are taking advantage of the enzymatic activity of FAP. With this strategy, a prodrug is administrated in an inactive form that is proteolytically activated by the *FAP* present in cancer activated fibroblasts localized in tumor microenvironment. Once activated, the drug targets any cell contained in the tumor [30,31]. Other therapies anti-stroma under development target integrins-extracellular membrane interactions [32,33] or target tumor stroma using T cells [34] or human mesenchymal stem cells [35,36]. Consequently is an active field of research and the identification of a high stroma subtype group of patients may be essential to obtain benefit from these treatments, administrating anti-stroma therapies just to this group of patients.

Since our survival results showed that Low-stroma-subtype identified lower risk patients and High-stroma-subtype and Mucinous-subtype identified higher risk patients, we contradict many reports indicating that MSI tumors have better clinical outcome than MSS/L tumors [37,38]. However, the Mucinous-subtype retains important factors usually found in poor prognostic tumors; a) mucinous tumors have worse clinical outcome and in our study mucinous and MSI tumors clustered together; b) high levels of *SPP1*, *FAP*, *GREMLIN1*, *CD55* or *REGIV* among others have been reported to be associated with cancer invasion, metastasis and poor prognostic in colon cancer [39-45]. These genes are up-regulated in clusters-3 and −4; c) the increased levels of *TFF2* and *MUC1*, characteristic of the Mucinous-subtype, have also been associated to a poor clinical outcome [46]; d) *BRAF* mutations have been shown as a worse prognostic factor [47,48]. Four out of the five MSI tumors in the Mucinous-subtype harbor *BRAF* mutations. For all there reasons, consequently, we could expect that patients of High-stroma-subtype and Mucinous-subtype had a worse clinical outcome.

The largest cluster was the Low-stroma-subtype and shows key clinical properties that specially distinguish this subtype from the other tumor subtypes. First, a 167 gene signature associated to this group of tumors distinguished low risk patients in an external clinical cohort. Second, eight different reported gene signatures including the extensively validated Oncotype-DX and ColoPrint [2-4,7-10,18], classified the Low-stroma-subtype patients in one group and the other tumor subtypes in a second group. Comparing microarray analysis across different studies and platforms is challenging. In general there is little or none overlapping among different gene signatures. In our study we found that eight different reported survival predictors and our 167 genes Low-stroma-subtype predictor, with almost no overlap among them, recognized the same group of patients in our data, the Low-stroma-subtype. Furthermore, our 167 genes Low-stroma-subtype predictor was able to identify in the external data set the patients with better clinical outcome. What is important and relevant for the application to the clinics is recognizing the same type of patients, not to demonstrate overlapping among different gene lists. This coincidence is important to confirm the potential of microarray gene expression for the identification of low risk patients. Nevertheless, it should be remarked that survival outcomes have not been confirmed with our own survival data and in the setting of a multivariable analysis. A higher sample size of homogeneous groups of patients will be necessary to establish the prognostic value of this molecular classification.

## Conclusions

With these findings, we propose a colon cancer classification in intrinsic molecular subtypes based on expression patterns. The novel colon tumor subtypes are associated to important clinicopathological features and show different survival times, but are not correlated to the histopathological stages. Tumor subtypes are established from initial tumor stages and validated in an external clinical cohort. Tumor microenvironment is important for the classification and for the malignant power of the tumor. Differential gene sets and biological pathways characterize each tumor subtype reflecting underlying mechanisms of carcinogenesis that may be used for the selection of targeted therapeutic procedures. The novel molecular classification reported in this study, may contribute to an improvement in the management of the patients with colorectal carcinoma and to a more comprehensive prognosis.

## Competing interests

The authors declare that they have no competing interests.

## Authors’ contributions

BPV, FMS and EDR supervised and designed the original study with the collaboration of TC. SHP, JALA and JSA performed the pathological study. ARL and BPV carried out the microarray experiments. ARL, BPV, GLC and FMS analyzed the data. CFP and ARL performed the statistical analysis. AC, JS and RA performed the selection and clinical study of the patients. All authors contributed to revising the article. BPV wrote the paper. All authors read and approved the final manuscript.

## Pre-publication history

The pre-publication history for this paper can be accessed here:

http://www.biomedcentral.com/1471-2407/12/260/prepub

## Supplementary Material

Additional file 1 Supplementary Information. Click here for file

Additional file 2 List of 1722 genes. Click here for file

Additional file 3 **Figure S1.** Hierarchical clustering of the combined set of 159 tumor samples. Click here for file

Additional file 4 Low-stroma-subtype 167 genes predictor. Click here for file
