# Supplementary figures and images for: Colon cancer molecular subtypes identified by expression profiling and associated to stroma, mucinous type and different clinical behavior
Source: BMC Cancer. 2012 Jun 19;12:260. doi: 10.1186/1471-2407-12-260 (PMC3571914; doi:10.1186/1471-2407-12-260)

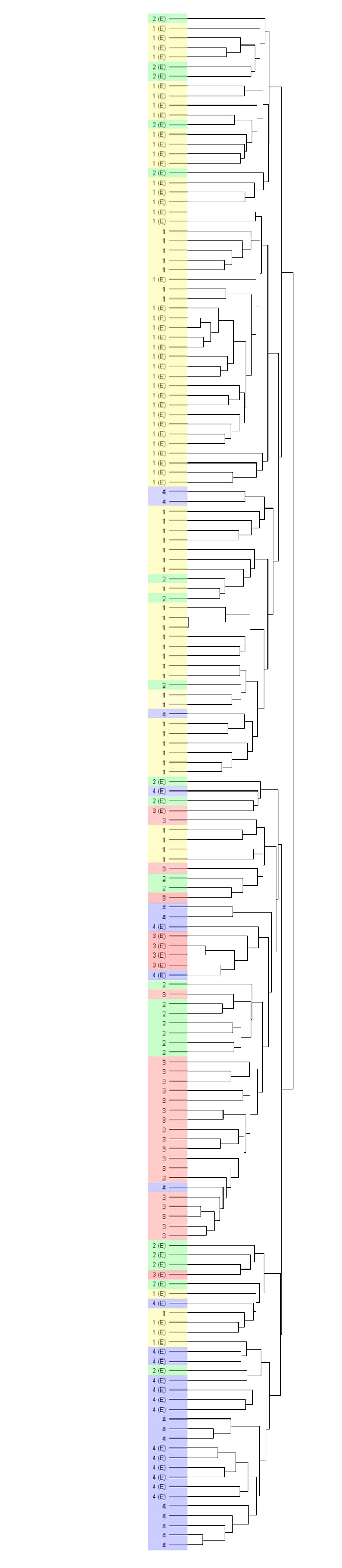

Supplement: Additional file 1 — Supplementary Information. [file 1471-2407-12-260-S1.tiff]
