# Supplementary material for: Colon cancer molecular subtypes identified by expression profiling and associated to stroma, mucinous type and different clinical behavior
Source: BMC Cancer. 2012 Jun 19;12:260. doi: 10.1186/1471-2407-12-260 (PMC3571914; doi:10.1186/1471-2407-12-260)
Supplement: Additional file 2 — List of 1722 genes. [file 1471-2407-12-260-S2.doc]

**Supplementary Information (Colon cancer molecular subtypes):**

**- Additional Methods:**

**- Analysis of Microsatellite instability**

**- Analysis of mutations in *KRAS* codon 12/13**

**- Analysis of *BRAF* V600E Mutation**

**- Analysis of *PI3K* Mutations**

**- Immunohistochemical analysis of β-catenin.**

**- Immunohistochemical analysis of Ki-67 (proliferation).**

**- Immunohistochemical analysis of M30 (apoptosis).**

**- Supplementary Tables:**

**- Table S1 Analysis of KEGG pathways**

**- Table S2 Correlations of tumor subtypes with clinical parameters**

**- Table S3 Performance of the classification of the 167 genes Low-stroma predictor**

**- Table S4 Coincident genes between different gene signatures**

**- Supplemental Legend to Figure S1**

**Additional Methods:**

**Analysis of Microsatellite instability**

DNA was purified from the same TRIZOL tumor lysate used for RNA purification once the RNA was already extracted. Sequences of Bat25, Bat26 and S5S346 were analyzed by PCR using the following primers:

Bat 26-sense: FAM-TGACTACTTTTGACTTCAGCC

Bat 26-antisense: AACCATTCAACATTTTTAACCC

Bat 25-sense: HEX-TCGCCTCCAAGAATGTAAGT

Bat-25-antisense: TCTGCATTTTAACTATGGCTC

S5S346-sense: TET-ACTCACTCTAGTGATAAATCGGG

S5S346-antisense: AGCAGATAAGACAAGTATTACTAGTT

Fragments were run in an ABI Prism 310 using the software ABI Prism Collection and Gene Scan 3.1 (Applied Biosystem). Samples with at least two microsatellites affected were considered as MSI+.

**Analysis of mutations in *KRAS* codon 12/13.**

Mutations in codon 12 and 13 of *KRAS* were analyzed by PCR followed by restriction analysis and electrophoresis following Hatzaki et al method (1) with some modifications:

For *KRAS* codon 12 PCR primers sequence were:

Sense: ACTGAATATAAACTTGTGGTAGTTGGACCT

Antisense: (FAM-6)-CCTCTATTGTTGGATCATATTCGTC

109 bp PCR product was treated with BstN1 to cut it in two pieces of 29 and 80 bp. Fragments were run in the ABI Prism 310. The 80 bp fragment was labeled with FAM-6 and was detected by the sequencer if there was wild type *KRAS*. If codon 12 was mutated restriction enzyme did not cut and the complete 109 PCR product was detected.

For *KRAS* codon 13 PCR primers sequence were:

Sense: (FAM-6) -TAACGCCTGCTGAAAATGACTG

Antisense: GTATCGTCAAGGCACTCTTGCCTAGG

79 bp PCR product was treated with HaeIII to cut it in two pieces of 53 and 26 bp. Fragments were run in the ABI Prism 3.10. The 53 bp fragment was labelled with FAM-6 and was detected by the sequencer if there was wild type *KRAS*. If codon 13 was mutated restriction enzyme did not cut and the complete 79 PCR product was detected

**Analysis of *BRAF* V600E Mutation:**

V600E *BRAF* Mutation was analyzed using Taq-Man Assays and Real Time-PCR (ABI Prism 7500 sequence detection system).

PCR primer and reporter sequences were:

*BRAF*-sense: 5’-CATGAAGACCTCACAGTAAAAATAGGTGAT-3’

*BRAF*-antisense: 5’-TGGGACCCACTCCATCGA-3’

Wild Type Reporter sequence: 5’-VIC-CTAGCTACAGTGAAATC-3’

Mutant Reporter sequence: 5’-FAM-TAGCTACAGAGAAATC-3’

Real-time PCR was performed in a final reaction volume of 25 μl containing 12.5 μl of 2× TaqMan Universal PCR Master Mix (Applied Biosystems), 0.625 μl of 40x Assay Mix and 20ng of DNA. Amplification conditions were 10 minutes at 95°C, followed by 40 cycles of 15 seconds at 92°C and 1 minute at 60°C.

**Analysis of *PI3K* Mutations:**

*PI3K* mutations in exon 9 and exon 20 were analyzed by PCR followed by direct sequencing.

PCR primer sequences were:

*PI3K*-Exon 9-sense: GCTTTTTCTGTAAATCATCTGTG

*PI3K*-Exon 9-antisense: CTGAGATCAGCCAAATTCAGT

*PI3K*-Exon 20-sense: ACATTCGAAAGACCCTAGCC

*PI3K*-Exon 20-antisense: CAATTCCTATGCAATCGGTCT

PCR was performed in a final volume of 25 μl using 50 ng of genomic DNA, 2.5 μl 10 × Taq buffer, 2.5 μl MgCl2 (25 mM), 0.5 μl of each primer (10 μM), 2.5 μl dNTP (2 mM) and 0.2 μl Taq polymerase (1 U/μl).

PCR conditions for *PI3K*-Exon 9 were: 10 min. at 95°C, followed by 10 cycles of 30 sec at 95°C, 30 sec at 60ºC and 30 sec at 72°C and 25 cycles of 30 sec at 95°C, 30 sec at 55ºC and 30 sec at 72°C. A final step of 10 min at 72ºC was added.

PCR amplification conditions for *PI3K*-Exon 20 were: 10 min. at 95°C, followed by 10 cycles of 30 sec at 95°C, 30 sec at 58ºC and 30 sec at 72°C and 25 cycles of 30 sec at 95°C, 30 sec at 55ºC and 30 sec at 72°C. A final step of 10 min at 72ºC was added.

Direct sequencing was carried out using BigDye terminator V 1.1 cycle sequencing kit (Applied Biosystems). Sequence analysis was performed in an ABI PRISM 3130. Primers used during sequencing were the same used for amplification.

**Immunohistochemical analysis of β-catenin.**

Sections 3 μm thick of TMAs were cut, deparaffinized in xylol, and then rehydrated in descending dilutions of ethanol. Antigen retrieval was done in a hot bath (100ºC) for 15 min with ER-1 Bond Retrieval solution (pH 6.0). The endogenous peroxidase activity was blocked by 10 min of incubation with H_2_O_2_ at room temperature. Sections were incubated with β-catenin polyclonal antibody (Master Diagnostica) during 60 min at room temperature. Then, sections were incubated with a secondary antibody (horseradish peroxidase (HRP)) for 30 min at room temperature followed by incubation with diamiobenzidine (DAB) for 5 min at room temperature. Tissues were counterstained with hematoxylin.

**Immunohistochemical analysis of Ki-67 (proliferation).**

Sections 3 μm thick of TMAs were cut, deparaffinized in xylol, and then rehydrated in descending dilutions of ethanol. Antigen retrieval was done in steamer with citrate buffer (pH 6.0) (Dako Real Target Retrieval solution) for 8 min. The endogenous peroxidase activity was blocked by 10 min of incubation with H_2_O_2_ at room temperature. Sections were incubated with ki67 monoclonal antibody (clone MIB1) (Dako) during 30 min at room temperature. Then, sections were incubated with a secondary antibody (horseradish peroxidase (HRP)) for 30 min at room temperature followed by incubation with diamiobenzidine (DAB) for 5 min at room temperature. Tissues were counterstained with hematoxylin.

**Immunohistochemical analysis of M30 (apoptosis).**

Sections 3 μm thick of TMAs were cut, deparaffinized in xylol, and then rehydrated in descending dilutions of ethanol. Antigen retrieval was done in a hot bath (100ºC) for 15 min with ER-1 Bond Retrieval solution (pH 6.0). The endogenous peroxidase activity was blocked by 10 min of incubation with H_2_O_2_ at room temperature. Sections were incubated with M30 Cytodeath monoclonal antibody (Roche Pharma) during 60 min at room temperature. Then, sections were incubated with a secondary antibody (horseradish peroxidase (HRP)) for 30 min at room temperature followed by incubation with diamiobenzidine (DAB) for 5 min at room temperature. Tissues were counterstained with hematoxylin.

1. Hatzaki A, Razi E, Anagnostopoulou K, et al: A modified mutagenic PCR-RFLP method for KRAS codon 12 and 13 mutations detection in NSCLC patients. *Mol Cell Probes* 2001; 15**:**243-247.

**Supplemental Tables:**

**Table S3 Performance of the 167 genes Low-stroma-subtype predictor**

| **Class** | **Sensitivity** | **Specificity** | **PPV** | **NPV** |
| --- | --- | --- | --- | --- |
| **Low-stroma** | 0.958 | 0.972 | 0.979 | 0.946 |
| **Other** | 0.972 | 0.958 | 0.946 | 0.979 |

Mean % of correct classification: 96%

PPV: Positive-Predictive Value, NPV: Negative-Predictive Value

**Table S4 Coincident genes between different gene signatures**

**Coincident genes (2) between Low-stroma predictor and Eschrich et al.**

NM_000582 Homo sapiens secreted phosphoprotein 1 (SPP1)

NM_173574 Homo sapiens zinc finger protein 683 (ZNF683)

**Coincident gene (1) between Low-stroma predictor and Garman et al. predictor:**

NM_006475 Homo sapiens periostin, osteoblast specific factor (POSTN)

**Coincident gene (1) between Low-stroma predictor and Wang et al. predictor:**

NM_004063 CDH17

**Coincident genes (12) between Low-stroma predictor and Jorissen et al. predictor:**

L12350 Human thrombospondin 2 (THBS2)

NM_000104 Homo sapiens cytochrome P450, (CYP1B1),

NM_000138 Homo sapiens fibrillin 1 (FBN1)

NM_000362 Homo sapiens TIMP metallopeptidase inhibitor 3 (TIMP3)

NM_000582 Homo sapiens secreted phosphoprotein 1 (SPP1), transcript variant 2

NM_002192 Homo sapiens inhibin, beta A (INHBA)

NM_003239 Homo sapiens transforming growth factor, beta 3 (TGFB3)

NM_004791 Homo sapiens integrin, beta-like 1 (ITGBL1)

NM_006475 Homo sapiens periostin, osteoblast specific factor (POSTN)

NM_006873 Homo sapiens stonin 1 (STON1)

NM_080927 Homo sapiens discoidin, CUB and LCCL domain containing 2 (DCBLD2),

NM_148672 Homo sapiens chemokine (C-C motif) ligand 28 (CCL28)

**Coincident genes (2) between Low-stroma predictor and Oncotype DX predictor:**

NM_004460 FAP

NM_002192 INHBA

**Coincident gene (1) between Low-stroma predictor and ColoPrint predictor:**

NM_000862 HSD3B1

**Legend to the supplemental figure**

**Figure S1** - **Hierarchical clustering of the combined set of 159 tumor samples**. Samples from cluster 5, normal tissue and adenomas were excluded. Yellow shadow: Cluster-1 or Low-stroma-subtype; green shadow: Cluster-2 or Immunoglobulin-related-subtype; red shadow: Cluster-3 or High-stroma-subtype; blue shadow: Cluster-4 or Mucinous-subtype; (E): Eschrich samples.
