# Supplementary material for: Colon cancer molecular subtypes identified by expression profiling and associated to stroma, mucinous type and different clinical behavior
Source: BMC Cancer. 2012 Jun 19;12:260. doi: 10.1186/1471-2407-12-260 (PMC3571914; doi:10.1186/1471-2407-12-260)
Supplement: Additional file 3 — Figure S1. Hierarchical clustering of the combined set of 159 tumor samples. [file 1471-2407-12-260-S3.pdf]

Order of genes in the heatmap	Unique id	UG cluster	Gene symbol	Map Location
1	THC2560976		THC2560976	
2	BC022881	Hs.652926	LOC644450	
3	NM_203414	Hs.417029	C17orf81	17p13.1
4	NM_178537	Hs.148074	B4GALNT4	11p15.5
5	NR_001564	Hs.529901	XIST	Xq13.2
6	BC020847	Hs.644600	LOC644246	17q21.31
7	NM_001828	Hs.889	CLC	19q13.2
8	NM_173549	Hs.171455	C8orf47	8q22.2
9	ENST00000302057	Hs.282089	IRX2	5p15.33
10	NM_002344	Hs.434481	LTK	15q15.1
11	NM_002220	Hs.2722	ITPKA	15q15.1
12	THC2536817		THC2536817	2q31.1
13	NM_172113	Hs.472877	EYA2	20q13.12
14	NM_032391	Hs.116467	PRAC	17q21.32
15	NM_006361	Hs.66731	HOXB13	17q21.32
16	NM_001884	Hs.591758	HAPLN1	5q14.3
17	NM_002054	Hs.516494	GCG	2q24.2
18	NM_001819	Hs.516874	CHGB	20p12.3
19	NM_017852	Hs.369279	NLRP2	19q13.42
20	NM_001012978	Hs.47209	NGFRAP1L1	Xq22.1
21	NM_006198	Hs.80296	PCP4	21q22.2
22	NM_005987	Hs.46320	SPRR1A	1q21.3
23	NM_005408	Hs.414629	CCL13	17q12
24	NM_001911	Hs.421724	CTSG	14q12
25	NM_024025	Hs.8719	DUSP26	8p12
26	NM_018286	Hs.173233	TMEM100	17q22
27	ENST00000370548		ENST00000370548	1p22.3
28	NM_006741	Hs.505662	PPP1R1A	12q13.2
29	NM_181789	Hs.526441	GLDN	15q21.2
30	NM_003381	Hs.53973	VIP	6q25.2
31	NM_001721	Hs.495731	BMX	Xp22.2
32	NM_005398	Hs.303090	PPP1R3C	10q23.32
33	NM_033014	Hs.109439	OGN	9q22.31
34	NM_004750	Hs.114948	CRLF1	19p13.11
35	NM_004181	Hs.518731	UCHL1	4p13
36	NM_000898	Hs.654473	MAOB	Xp11.3
37	NM_003012	Hs.213424	SFRP1	8p11.21
38	NM_006307	Hs.15154	SRPX	Xp11.4
39	NM_198289	Hs.249129	CIDEA	18p11.21
40	NM_007177	Hs.506357	FAM107A	3p14.2
41	AF348994	Hs.513626	MT1JP	16q13
42	NM_153370	Hs.25391	PI16	6p21.2
43	NM_000261	Hs.436037	MYOC	1q24.3
44	NM_024730	Hs.115497	FLJ22655	12p12.3
45	NM_138290	Hs.411488	RPIB9	7q21.12
46	NM_002606	Hs.473927	PDE9A	21q22.3
47	AK000158	Hs.210995	CA12	15q22.2
48	NM_004816	Hs.118003	C9orf61	9q21.11
49	NM_003759	Hs.5462	SLC4A4	4q13.3

50	NM_000067	Hs.155097	CA2	8q21.2
51	NM_006846	Hs.331555	SPINK5	5q33.1
52	NM_014312	Hs.112377	VSIG2	11q24.2
53	AK124946	Hs.162868	DPF3	14q24.2
54	NM_017682	Hs.435611	BEST2	19p13.13
55	ENST00000371483		GPR120	10q23.33
56	NM_006615	Hs.498021	CAPN9	1q42.2
57	THC2721785		THC2721785	1p33
58	THC2559929		THC2559929	14q32.33
59	NM_002614	Hs.444751	PDZK1	1q21.1
60	NM_152672	Hs.651164	OSTalpha	3q29
61	NM_000862	Hs.364941	HSD3B1	1p12
62	NM_005123	Hs.282735	NR1H4	12q23.1
63	NM_173625	Hs.439154	C17orf78	17q12
64	NM_176782	Hs.554944	C1orf179	1p32.3
65	NM_001169	Hs.176658	AQP8	16p12.1
66	NM_001275	Hs.150793	CHGA	14q32.12
67	NM_004827	Hs.480218	ABCG2	4q22.1
68	NM_004160	Hs.169249	PYY	17q21.31
69	NM_005478	Hs.251380	INSL5	1p31.3
70	NM_017716	Hs.272789	MS4A12	11q12.2
71	A_32_P157213		A_32_P157213	19q13.31
72	NM_033553	Hs.778	GUCA2A	1p34.2
73	NM_012128	Hs.567422	CLCA4	1p22.3
74	AI659667	Hs.516105	AI659667	2p13.1
75	NM_139250	Hs.559536	CTAG1A	Xq28
76	NM_000870	Hs.483773	HTR4	5q33.1
77	A_32_P171043		A_32_P171043	18q21.32
78	NM_002196	Hs.89584	INSM1	20p11.23
79	THC2686967		THC2686967	17q25.1
80	NM_017878	Hs.272805	HRASLS2	11q12.3
81	NM_020973	Hs.653107	GBA3	4p15.31
82	NM_006919	Hs.227948	SERPINB3	18q21.33
83	A_32_P166653		A_32_P166653	1q41
84	CB959193	Hs.97644	CB959193	11q12.3
85	NM_006998	Hs.116428	SCGN	6p22.2
86	NM_021637	Hs.45140	TMEM35	Xq22.1
87	NM_000717	Hs.89485	CA4	17q23.1
88	NM_001738	Hs.23118	CA1	8q21.2
89	NM_007102	Hs.32966	GUCA2B	1p34.2
90	AK124400	Hs.652438	LOC553137	6q21
91	NM_022843	Hs.391781	PCDH20	13q21.31
92	NM_000112	Hs.302738	SLC26A2	5q33.1
93	NM_000111	Hs.1650	SLC26A3	7q31.1
94	NM_178859	Hs.534533	OSTbeta	15q22.31
95	NM_002010	Hs.111	FGF9	13q12.11
96	NM_014033	Hs.655369	METTL7A	12q13.13
97	NM_145259	Hs.352338	ACVR1C	2q24.1
98	AF435956	Hs.591566	FAM82A	2p22.2
99	NM_173833	Hs.591833	SCARA5	8p21.1
100	NM_000667	Hs.654433	ADH1A	4q23
101	NM_000669	Hs.654537	ADH1C	4q23

102	BC033223	Hs.372492	SLC45A4	8q24.3
103	NM_002885	Hs.148178	RAP1GAP	1p36.12
104	NM_001012506	Hs.476399	CCDC66	3p14.3
105	BX119852	Hs.258100	BX119852	2p25.2
106	AA479896	Hs.587119	AA479896	20p12.3
107	NM_013347	Hs.659349	RPA4	Xq21.33
108	CV575560	Hs.659326	CV575560	17p11.2
109	NM_020857	Hs.23876	VPS18	15q15.1
110	NM_182527	Hs.643608	CABP7	22q12.2
111	THC2579650		THC2579650	15q26.1
112	THC2609820		THC2609820	8p21.1
113	THC2541992		THC2541992	1q32.1
114	NM_005853	Hs.435730	IRX5	16q12.2
115	CB162722	Hs.667977	CB162722	3p25.2
116	NM_018047	Hs.202023	RBM22	5q33.1
117	NM_014931	Hs.515610	SAPS1	19q13.42
118	NM_024512	Hs.657345	LRRC2	3p21.31
119	NM_014308	Hs.278901	PIK3R5	17p13.1
120	THC2672701		THC2672701	17q25.3
121	ENST00000314088	Hs.622290	HIST1H2AC	6p22.1
122	NM_005319	Hs.7644	HIST1H1C	6p22.1
123	A_23_P170713		A_23_P170713	6p22.1
124	NM_006980	Hs.532216	MTERF	7q21.2
125	CR933606	Hs.653099	C20orf80	20q11.1
126	NM_002252	Hs.414489	KCNS3	2p24.2
127	NM_000313	Hs.64016	PROS1	3q11.2
128	CR627475	Hs.125038	FAM92A1	8q22.1
129	NM_018476	Hs.334370	BEX1	Xq22.1
130	NM_032621	Hs.398989	BEX2	Xq22.1
131	NM_025176	Hs.631508	RP4-691N24.1	20p11.21
132	ENST00000372602	Hs.496530	TMSL8	Xq22.2
133	NM_052969	Hs.647900	RPL39L	3q27.3
134	NM_020685	Hs.47166	C3orf14	3p14.2
135	NM_014766	Hs.520740	SCRN1	7p15.1
136	NM_001454	Hs.651204	FOXJ1	17q25.1
137	NM_024532	Hs.602792	SPAG16	2q34
138	NR_002166	Hs.446620	SEDLP	19q13.43
139	NM_021632	Hs.407694	ZNF350	19q13.33
140	NM_198542	Hs.579576	ZNF773	19q13.43
141	NM_005773	Hs.596242	ZNF256	19q13.43
142	NM_023926	Hs.235390	ZSCAN18	19q13.43
143	NM_022804	Hs.564847	SNURF	15q11.2
144	NM_004052	Hs.144873	BNIP3	10q26.3
145	NM_016073	Hs.513954	HDGFRP3	15q25.2
146	THC2644897		THC2644897	12p12.2
147	NM_000484	Hs.693586	APP	21q21.3
148	NM_152718	Hs.60640	VWCE	11q12.2
149	NM_025135	Hs.436636	FHOD3	18q12.2
150	NM_016848	Hs.656806	SHC3	9q22.1
151	NM_014344	Hs.39384	FJX1	11p13
152	NM_182898	Hs.437075	CREB5	7p15.1
153	NM_003102	Hs.2420	SOD3	4p15.2

154	NM_007283	Hs.277035	MGLL	3q21.3
155	NM_020182	Hs.517155	TMEPAI	20q13.31
156	NM_003043	Hs.529488	SLC6A6	3p25.1
157	NM_000358	Hs.369397	TGFBI	5q31.2
158	ENST00000382327	Hs.156727	ANKH	5p15.2
159	NM_005978	Hs.516484	S100A2	1q21.3
160	NM_198129	Hs.436367	LAMA3	18q11.2
161	NM_018891	Hs.591484	LAMC2	1q25.3
162	NM_005562	Hs.591484	LAMC2	1q25.3
163	NM_002423	Hs.2256	MMP7	11q22.2
164	NM_014220	Hs.351316	TM4SF1	3q25.1
165	NM_020130	Hs.591849	C8orf4	8p11.21
166	NM_018948	Hs.605445	ERRFI1	1p36.23
167	NM_003897	Hs.655404	IER3	6p21.33
168	AL832183	Hs.436426	LOC284454	19p13.12
169	NM_006186	Hs.563344	NR4A2	2q24.1
170	NM_005252	Hs.25647	FOS	14q24.3
171	NM_004024	Hs.460	ATF3	1q32.3
172	NM_002135	Hs.524430	NR4A1	12q13.13
173	NM_001964	Hs.326035	EGR1	5q31.2
174	NM_005213	Hs.518198	CSTA	3q21.1
175	NM_000032	Hs.522666	ALAS2	Xp11.21
176	NM_000518	Hs.654454	HBB	11p15.4
177	NM_000519	Hs.523443	HBD	11p15.4
178	A_24_P147849		A_24_P147849	
179	NM_000517	Hs.654744	HBA2	16p13.3
180	NM_000558	Hs.449630	HBA1	16p13.3
181	NM_006512	Hs.654493	SAA4	11p15.1
182	NM_030754	Hs.654517	SAA2	11p15.1
183	NM_000331	Hs.632144	SAA1	11p15.1
184	NM_031419	Hs.319171	NFKBIZ	3q12.3
185	NM_005204	Hs.432453	MAP3K8	10p11.23
186	NM_005746	Hs.489615	PBEF1	7q22.2
187	A_24_P592591		A_24_P592591	10p11.21
188	NM_004591	Hs.75498	CCL20	2q36.3
189	NM_001511	Hs.789	CXCL1	4q13.3
190	NM_002089	Hs.590921	CXCL2	4q13.3
191	NM_002090	Hs.89690	CXCL3	4q13.3
192	NM_032895	Hs.597755	MGC14376	17p13.3
193	AY358804	Hs.505141	AY358804	12p11.21
194	NM_002160	Hs.143250	TNC	9q33.1
195	NM_002658	Hs.77274	PLAU	10q22.2
196	NM_006474	Hs.468675	PDPN	1p36.21
197	NM_003392	Hs.643085	WNT5A	3p14.3
198	NM_002422	Hs.375129	MMP3	11q22.2
199	NM_002421	Hs.83169	MMP1	11q22.2
200	NM_002994	Hs.89714	CXCL5	4q13.3
201	NM_002425	Hs.2258	MMP10	11q22.2
202	NM_000640	Hs.336046	IL13RA2	Xq23
203	NM_000600	Hs.654458	IL6	7p15.3
204	NM_000963	Hs.196384	PTGS2	1q31.1
205	NM_006528	Hs.438231	TFPI2	7q21.3

206	NM_000641	Hs.467304	IL11	19q13.42
207	NM_000854	Hs.654462	GSTT2	22q11.23
208	AA554330	Hs.600066	AA554330	18q21.1
209	NM_002200	Hs.521181	IRF5	7q32.1
210	U52054	Hs.561411	U52054	1p11.2
211	NM_002436	Hs.496984	MPP1	Xq28
212	NM_006646	Hs.635221	WASF3	13q12.13
213	NM_005410	Hs.275775	SEPP1	5p12
214	NM_003059	Hs.310591	SLC22A4	5q31.1
215	THC2668815		THC2668815	9q34.11
216	NM_003462	Hs.406050	DNALI1	1p34.3
217	THC2623335		THC2623335	16q12.1
218	BC031342	Hs.445239	BC031342	7p15.2
219	NM_003063	Hs.334629	SLN	11q22.3
220	U10991	Hs.502266	C11orf41	11p13
221	BC040542	Hs.650822	KIAA0802	18p11.22
222	NM_001013642	Hs.355747	LOC388610	1p36.11
223	NM_053001	Hs.253247	OSR2	8q22.2
224	NM_004282	Hs.55220	BAG2	6p12.1
225	NM_005855	Hs.471783	RAMP1	2q37.3
226	NM_002155	Hs.654614	HSPA6	1q23.3
227	NM_005345	Hs.520028	HSPA1A	6p21.33
228	NM_005527	Hs.690634	HSPA1L	6p21.33
229	NM_001174	Hs.435291	ARHGAP6	Xp22.2
230	NM_053277	Hs.693698	CLIC6	21q22.12
231	NM_021012	Hs.200629	KCNJ12	17p11.2
232	NM_004962	Hs.2171	GDF10	10q11.22
233	NM_000129	Hs.335513	F13A1	6p25.1
234	BX097190	Hs.7413	BX097190	3q23
235	NM_133631	Hs.13640	ROBO1	3p12.3
236	NM_016315	Hs.470887	GULP1	2q32.2
237	NM_002091	Hs.153444	GRP	18q21.32
238	A_32_P131449		A_32_P131449	5q33.1
239	NM_182943	Hs.477866	PLOD2	3q24
240	BC044246	Hs.591341	KIAA1913	6q23.1
241	NM_001873	Hs.75360	CPE	4q32.3
242	NM_007029	Hs.521651	STMN2	8q21.13
243	S82024		STMN2	8q21.13
244	NM_006744	Hs.50223	RBP4	10q23.33
245	AK124396	Hs.633447	AK124396	4q31.22
246	BC092429	Hs.660885	BC092429	17p13.1
247	NM_183376	Hs.6093	ARRDC4	15q26.3
248	NM_006472	Hs.533977	TXNIP	1q21.1
249	NM_006274	Hs.50002	CCL19	9p13.3
250	NM_004867	Hs.17109	ITM2A	Xq21.1
251	NM_001928	Hs.155597	CFD	19p13.3
252	NM_004167	Hs.656286	CCL15	17q12
253	NM_003278	Hs.476092	CLEC3B	3p21.31
254	NM_001932	Hs.396566	MPP3	17q21.31
255	NM_207380	Hs.32433	FLJ43339	15q15.1
256	NM_003417	Hs.515634	ZNF264	19q13.43
257	NM_017870	Hs.118552	TMEM132A	11q12.2

258	NM_000152	Hs.1437	GAA	17q25.3
259	NM_015326	Hs.497575	SRGAP2	1q32.1
260	NM_006885	Hs.652666	ATBF1	16q22.3
261	AK129956	Hs.170131	AK129956	19p13.3
262	NM_003507	Hs.173859	FZD7	2q33.1
263	NM_032780	Hs.564188	TMEM25	11q23.3
264	NM_014380	Hs.448588	NGFRAP1	Xq22.2
265	NM_016269	Hs.555947	LEF1	4q25
266	NM_000076	Hs.106070	CDKN1C	11p15.4
267	NM_001546	Hs.519601	ID4	6p22.3
268	NM_000237	Hs.180878	LPL	8p21.3
269	NM_002514	Hs.235935	NOV	8q24.12
270	AJ710526	Hs.595068	AJ710526	10q23.33
271	NM_016606	Hs.416090	REEP2	5q31.2
272	NM_194272	Hs.436518	RBPMS2	15q22.31
273	NM_000702	Hs.34114	ATP1A2	1q23.2
274	NM_003248	Hs.211426	THBS4	5q14.1
275	NM_000743	Hs.89605	CHRNA3	15q25.1
276	NM_002247	Hs.144795	KCNMA1	10q22.3
277	NM_015424	Hs.432379	CHRDL2	11q13.4
278	NM_032105	Hs.444403	PPP1R12B	1q32.1
279	AL137734	Hs.437437	DKFZp586C0721	5q33.1
280	NM_004137	Hs.484099	KCNMB1	5q35.1
281	AL833294	Hs.655519	SYNPO2	4q26
282	NM_001615	Hs.516105	ACTG2	2p13.1
283	NM_001299	Hs.465929	CNN1	19p13.2
284	NM_053025	Hs.556600	MYLK	3q21.1
285	NM_002474	Hs.460109	MYH11	16p13.11
286	NM_022844	Hs.460109	MYH11	16p13.11
287	NM_001927	Hs.594952	DES	2q35
288	NM_145728	Hs.207106	DMN	15q26.3
289	NM_203339	Hs.436657	CLU	8p21.1
290	NM_022370	Hs.435621	ROBO3	11q24.2
291	NM_002145	Hs.514289	HOXB2	17q21.32
292	NM_006622	Hs.398157	PLK2	5q11.2
293	NM_080927	Hs.203691	DCBLD2	3q12.1
294	NM_014945	Hs.49688	ABLIM3	5q33.1
295	NM_018043	Hs.503074	TMEM16A	11q13.3
296	NM_031866	Hs.302634	FZD8	10p11.21
297	ENST00000367013	Hs.446946	C1orf133	1q32.2
298	NM_078487	Hs.72901	CDKN2B	9p21.3
299	DB381305	Hs.629427	DB381305	18q21.33
300	NM_152852	Hs.523702	MS4A6A	11q12.1
301	NM_001080425	Hs.184736	BEXL1	Xq22.1
302	NM_021939	Hs.463035	FKBP10	17q21.2
303	NM_002961	Hs.654444	S100A4	1q21.3
304	NM_001423	Hs.693682	EMP1	12p13.1
305	NM_000700	Hs.494173	ANXA1	9q21.13
306	NM_001008540	Hs.593413	CXCR4	2q21.3
307	NM_002922	Hs.75256	RGS1	1q31.2
308	NM_005627	Hs.510078	SGK	6q23.2
309	NM_006732	Hs.590958	FOSB	19q13.32

310	NM_002928	Hs.413297	RGS16	1q25.3
311	THC2516687	Hs.8867	THC2516687	1p22.3
312	NM_000399	Hs.1395	EGR2	10q21.2
313	NM_001901	Hs.591346	CTGF	6q23.2
314	NM_001554	Hs.8867	CYR61	1p22.3
315	NM_001781	Hs.208854	CD69	12p13.31
316	NM_004417	Hs.171695	DUSP1	5q35.1
317	NM_002923	Hs.78944	RGS2	1q31.2
318	NM_004951	Hs.784	EBI2	13q32.3
319	NM_005261	Hs.654463	GEM	8q22.1
320	ENST00000339446	Hs.530443	LOC387763	11p11.2
321	NM_006988	Hs.643357	ADAMTS1	21q21.3
322	NM_173198	Hs.279522	NR4A3	9q31.1
323	NM_006108	Hs.654637	SPON1	11p15.2
324	NM_001080393	Hs.431092	GLT8D4	3p13
325	AW961597	Hs.675553	AW961597	3p13
326	THC2515368		THC2515368	3p13
327	NM_021020	Hs.521432	LZTS1	8p21.3
328	NM_014571	Hs.472566	HEYL	1p34.2
329	AK023854	Hs.526594	OBSL1	2q35
330	NM_024563	Hs.13528	C5orf23	5p13.3
331	NM_030762	Hs.177841	BHLHB3	12p12.1
332	NM_181847	Hs.121520	AMIGO2	12q13.11
333	NM_000693	Hs.459538	ALDH1A3	15q26.3
334	NM_000014	Hs.212838	A2M	12p13.31
335	AL833309	Hs.622596	AL833309	3q26.2
336	NM_001003793	Hs.221436	RBMS3	3p24.1
337	NM_003277	Hs.505337	CLDN5	22q11.21
338	NM_004684	Hs.62886	SPARCL1	4q22.1
339	NM_021219	Hs.517227	JAM2	21q21.3
340	NM_021914	Hs.180141	CFL2	14q13.2
341	NM_015687	Hs.526972	FILIP1	6q14.1
342	NM_032784	Hs.135254	RSPO3	6q22.33
343	NM_024769	Hs.591949	ASAM	11q24.1
344	AK074473	Hs.559353	C20orf82	20p12.1
345	NM_004791	Hs.584836	ITGBL1	13q33.1
346	NM_000095	Hs.1584	COMP	19p13.11
347	NM_005940	Hs.143751	MMP11	22q11.23
348	NM_003326	Hs.181097	TNFSF4	1q25.1
349	NM_000493	Hs.520339	COL10A1	6q22.1
350	BF337308	Hs.619396	BF337308	
351	NM_007021	Hs.93675	C10orf10	10q11.21
352	NM_058229	Hs.403933	FBXO32	8q24.13
353	AK023391	Hs.592775	AK023391	8q24.13
354	NM_022343	Hs.493819	C9orf19	9p13.3
355	A_23_P421323		A_23_P421323	
356	NM_052966	Hs.518662	FAM129A	1q25.3
357	NM_005909	Hs.584777	MAP1B	5q13.2
358	AK055112	Hs.637017	AK055112	5q13.2
359	NM_015833	Hs.474018	ADARB1	21q22.3
360	NM_015103	Hs.301685	PLXND1	3q21.3
361	NM_003062	Hs.604116	SLIT3	5q35.1

362	NM_000609	Hs.522891	CXCL12	10q11.21
363	NM_012261	Hs.22920	C20orf103	20p12.2
364	NM_173653	Hs.302257	SLC9A9	3q24
365	NM_024692	Hs.122927	CLIP4	2p23.2
366	NM_199072	Hs.427236	MDFIC	7q31.2
367	NM_002113	Hs.363396	CFHR1	1q31.3
368	NM_021023	Hs.575869	CFHR3	1q31.3
369	NM_001014975	Hs.363396	CFH	1q31.3
370	NM_003881	Hs.592145	WISP2	20q13.12
371	NM_000618	Hs.160562	IGF1	12q23.2
372	W60781	Hs.391561	W60781	8q21.13
373	ENST00000372045	Hs.496587	ENST00000372045	Xq22.3
374	A_32_P210193		A_32_P210193	12p12.3
375	NM_003613	Hs.442180	CILP	15q22.31
376	NM_000900	Hs.365706	MGP	12p12.3
377	NM_000961	Hs.302085	PTGIS	20q13.13
378	AK055387	Hs.21639	SPEG	2q35
379	NM_002667	Hs.170839	PLN	6q22.31
380	AK129844	Hs.170839	PLN	6q22.31
381	NM_145056	Hs.515490	DACT3	19q13.32
382	AI042308	Hs.666842	AI042308	11q23.3
383	NM_213674	Hs.300772	TPM2	9p13.3
384	NM_003734	Hs.198241	AOC3	17q21.31
385	NM_181526	Hs.504687	MYL9	20q11.23
386	NM_001613	Hs.500483	ACTA2	10q23.31
387	NM_001001522	Hs.632099	TAGLN	11q23.3
388	NM_004787	Hs.29802	SLIT2	4p15.31
389	AW268902	Hs.29802	AW268902	4p15.31
390	NM_198148	Hs.656887	CPXM2	10q26.13
391	NM_002048	Hs.65029	GAS1	9q21.33
392	NM_199168	Hs.522891	CXCL12	10q11.21
393	NM_003480	Hs.512842	MFAP5	12p13.31
394	NM_000104	Hs.154654	CYP1B1	2p22.2
395	NM_003013	Hs.481022	SFRP2	4q31.3
396	NM_003239	Hs.592317	TGFB3	14q24.3
397	BE835321	Hs.592317	BE835321	14q24.3
398	NM_199511	Hs.477128	CCDC80	3q13.2
399	NM_006207	Hs.458573	PDGFRL	8p22
400	NM_003485	Hs.8882	GPR68	14q32.12
401	NM_001039580	Hs.61271	MAP9	4q32.1
402	NM_002023	Hs.519168	FMOD	1q32.1
403	NM_206943	Hs.654497	LTBP1	2p22.3
404	AL049443	Hs.660870	AL049443	2p16.3
405	NM_000064	Hs.529053	C3	19p13.3
406	A_24_P561165		A_24_P561165	11q12.1
407	NM_005099	Hs.211604	ADAMTS4	1q23.3
408	NM_212482	Hs.203717	FN1	2q35
409	NM_170744	Hs.585457	UNC5B	10q22.1
410	NM_001001396	Hs.343522	ATP2B4	1q32.1
411	AK024680	Hs.660596	AK024680	2q33.3
412	NM_004370	Hs.101302	COL12A1	6q13
413	NM_138440	Hs.372579	VASN	16p13.3

414	NM_012445	Hs.302963	SPON2	4p16.3
415	NM_007361	Hs.369840	NID2	14q22.1
416	AB058761	Hs.54925	ZNF469	16q24.2
417	NM_001849	Hs.420269	COL6A2	21q22.3
418	NM_001848	Hs.474053	COL6A1	21q22.3
419	Z74615	Hs.172928	COL1A1	17q21.33
420	NM_002609	Hs.509067	PDGFRB	5q33.1
421	NM_001846	Hs.508716	COL4A2	13q34
422	AW138903	Hs.8546	AW138903	19p13.12
423	BX427588	Hs.669724	BX427588	7q21.3
424	NM_002889	Hs.647064	RARRES2	7q36.1
425	NM_013372	Hs.40098	GREM1	15q13.3
426	BC039414	Hs.655414	BC039414	4q32.1
427	NM_002404	Hs.296049	MFAP4	17p11.2
428	NM_005269	Hs.632702	GLI1	12q13.3
429	NM_004349	Hs.368431	RUNX1T1	8q21.3
430	ENST00000377047	Hs.444329	GPC6	13q32.1
431	NM_006486	Hs.24601	FBLN1	22q13.31
432	NM_001734	Hs.458355	C1S	12p13.31
433	NM_004598	Hs.654695	SPOCK1	5q31.2
434	NM_181435	Hs.171929	C1QTNF3	5p13.3
435	BC020879	Hs.664877	MGC24103	9p22.3
436	NR_002766	Hs.525589	MEG3	14q32.2
437	NM_014476	Hs.85862	PDLIM3	4q35.1
438	NM_005086	Hs.183428	SSPN	12p12.1
439	NM_004105	Hs.76224	EFEMP1	2p16.1
440	NM_153026	Hs.524348	PRICKLE1	12q12
441	NM_006873	Hs.44385	STON1	2p16.3
442	AK095791	Hs.411391	AK095791	11q24.1
443	NM_001856	Hs.368921	COL16A1	1p35.2
444	NM_016651	Hs.48950	DACT1	14q23.1
445	NM_002192	Hs.583348	INHBA	7p14.1
446	NM_014112	Hs.657018	TRPS1	8q23.3
447	NM_032812	Hs.658134	PLXDC2	10p12.31
448	NM_130386	Hs.464422	COLEC12	18p11.32
449	NM_053044	Hs.479119	HTRA3	4p16.1
450	NM_001004019	Hs.198862	FBLN2	3p25.1
451	NM_024893	Hs.124638	C20orf39	20p11.21
452	NM_000362	Hs.644633	TIMP3	22q12.3
453	NM_002615	Hs.532768	SERPINF1	17p13.3
454	NM_005576	Hs.65436	LOXL1	15q24.1
455	NM_144601	Hs.298198	CMTM3	16q22.1
456	NM_003380	Hs.642813	VIM	10p12.33
457	THC2574606		THC2574606	10p12.33
458	NM_020856	Hs.278436	TSHZ3	19q12
459	ENST00000375377	Hs.533953	KIAA1462	10p11.23
460	NM_012232	Hs.437191	PTRF	17q21.31
461	NM_007085	Hs.269512	FSTL1	3q13.33
462	NM_033138	Hs.490203	CALD1	7q33
463	NM_000393	Hs.445827	COL5A2	2q32.2
464	NM_000138	Hs.591133	FBN1	15q21.1
465	NM_000089	Hs.489142	COL1A2	7q21.3

466	NM_000090	Hs.443625	COL3A1	2q32.2
467	NM_003118	Hs.111779	SPARC	5q33.1
468	NM_006169	Hs.503911	NNMT	11q23.2
469	NM_005110	Hs.30332	GFPT2	5q35.3
470	NM_016205	Hs.570855	PDGFC	4q32.1
471	NM_002775	Hs.501280	HTRA1	10q26.13
472	NM_023110	Hs.264887	FGFR1	8p12
473	NM_000168	Hs.21509	GLI3	7p14.1
474	NM_001129	Hs.439463	AEBP1	7p13
475	NM_003014	Hs.658169	SFRP4	7p14.1
476	NM_005202	Hs.353001	COL8A2	1p34.3
477	NM_001850	Hs.654548	COL8A1	3q12.1
478	AL359062	Hs.134830	AL359062	3q12.1
479	NM_015170	Hs.409602	SULF1	8q13.3
480	L12350	Hs.371147	THBS2	6q27
481	NM_032532	Hs.520525	FNDC1	6q25.3
482	NM_001797	Hs.116471	CDH11	16q21
483	NM_032208	Hs.165859	ANTXR1	2p14
484	NM_005504	Hs.438993	BCAT1	12p12.1
485	NM_006475	Hs.136348	POSTN	13q13.3
486	NM_006868	Hs.99528	RAB31	18p11.22
487	NM_138455	Hs.405614	CTHRC1	8q22.3
488	NM_004460	Hs.654370	FAP	2q24.2
489	NM_017680	Hs.435655	ASPN	9q22.31
490	NM_000396	Hs.632466	CTSK	1q21.2
491	NM_001920	Hs.156316	DCN	12q21.33
492	NM_002345	Hs.406475	LUM	12q21.33
493	NM_152330	Hs.434914	FRMD6	14q22.1
494	NM_003596	Hs.421194	TPST1	7q11.21
495	NM_016206	Hs.435013	VGLL3	3p12.1
496	NM_030786	Hs.655938	SYNC1	1p35.1
497	BM999343	Hs.99528	BM999343	18p11.22
498	NM_004530	Hs.513617	MMP2	16q12.2
499	NM_003247	Hs.371147	THBS2	6q27
500	NM_001030059	Hs.40479	PPAPDC1A	10q26.12
501	NM_004403	Hs.520708	DFNA5	7p15.3
502	NM_018004	Hs.658956	TMEM45A	3q12.2
503	NM_003254	Hs.522632	TIMP1	Xp11.23
504	NM_014583	Hs.475353	LMCD1	3p26.1
505	NM_032849	Hs.646647	C13orf33	13q12.3
506	AY098593	Hs.439341	KGFLP1	9p11.2
507	NM_004995	Hs.2399	MMP14	14q11.2
508	NM_178234	Hs.591845	TUSC3	8p22
509	NM_006832	Hs.509343	PLEKHC1	14q22.2
510	NM_000165	Hs.74471	GJA1	6q22.31
511	AF200348	Hs.332197	PXDN	2p25.3
512	NM_016307	Hs.660115	PRRX2	9q34.11
513	AW276332	Hs.406475	AW276332	12q21.33
514	NM_002998	Hs.1501	SDC2	8q22.1
515	AK022110	Hs.289044	AK022110	5q11.2
516	NM_031442	Hs.8769	TMEM47	Xp21.1
517	NM_018357	Hs.416755	LARP6	15q23

518	NM_005725	Hs.310458	TSPAN2	1p13.2
519	NM_001033564	Hs.591340	LOC619208	6q21
520	NM_005711	Hs.482730	EDIL3	5q14.3
521	NM_004348	Hs.535845	RUNX2	6p12.3
522	NM_013409	Hs.9914	FST	5q11.2
523	NM_016613	Hs.567498	C4orf18	4q32.1
524	AK025997	Hs.655087	MCTP1	5q15
525	NM_000565	Hs.591492	IL6R	1q21.3
526	AB002384	Hs.559459	C6orf32	6p22.2
527	NM_021615	Hs.655622	CHST6	16q23.1
528	NM_015184	Hs.202010	PLCL2	3p24.3
529	NM_001874	Hs.654387	CPM	12q15
530	NM_005949	Hs.513626	MT1F	16q13
531	NM_176870	Hs.647370	MT1M	16q13
532	NM_005953	Hs.647371	MT2A	16q13
533	ENST00000245185	Hs.647371	MT2A	16q13
534	NM_005951	Hs.438462	MT1H	16q13
535	NM_175617	Hs.534330	MT1E	16q13
536	NM_005952	Hs.374950	MT1X	16q13
537	X97261	Hs.647358	MT1L	16q13
538	NM_005950	Hs.433391	MT1G	16q13
539	NM_005947	Hs.656629	MT1B	16q13
540	NM_000450	Hs.89546	SELE	1q24.2
541	NM_000552	Hs.440848	VWF	12p13.31
542	NM_005282	Hs.17170	GPR4	19q13.32
543	NM_018371	Hs.655166	ChGn	8p21.3
544	NM_170736	Hs.411299	KCNJ15	21q22.13
545	AA593970	Hs.608251	AA593970	2p14
546	NM_001124	Hs.441047	ADM	11p15.4
547	NM_139314	Hs.9613	ANGPTL4	19p13.2
548	A_23_P170719		A_23_P170719	19p13.2
549	NM_001109	Hs.501574	ADAM8	10q26.3
550	NM_138931	Hs.478588	BCL6	3q27.3
551	A_23_P123234		A_23_P123234	7q22.1
552	BC060766	Hs.655169	SLC2A14	12p13.31
553	NM_000201	Hs.643447	ICAM1	19p13.2
554	NM_006291	Hs.525607	TNFAIP2	14q32.32
555	NM_002133	Hs.517581	HMOX1	22q12.3
556	NM_145699	Hs.348983	APOBEC3A	22q13.1
557	NM_173842	Hs.81134	IL1RN	2q13
558	NM_006018	Hs.458425	GPR109B	12q24.31
559	NM_021935	Hs.528665	PROK2	3p13
560	NM_000576	Hs.126256	IL1B	2q13
561	NM_000584	Hs.624	IL8	4q13.3
562	NM_007115	Hs.437322	TNFAIP6	2q23.3
563	NM_018643	Hs.283022	TREM1	6p21.1
564	NM_002984	Hs.75703	CCL4	17q12
565	D00044	Hs.514107	CCL3	17q12
566	NM_003955	Hs.527973	SOCS3	17q25.3
567	NM_020530	Hs.248156	OSM	22q12.2
568	NM_015714	Hs.432132	G0S2	1q32.2
569	NM_000636	Hs.487046	SOD2	6q25.3

570	NM_002965	Hs.112405	S100A9	1q21.3
571	NM_002432	Hs.153837	MNDA	1q23.1
572	NM_016184	Hs.504657	CLEC4A	12p13.31
573	NM_014358	Hs.236516	CLEC4E	12p13.31
574	NM_002664	Hs.468840	PLEK	2p14
575	NM_005565	Hs.304475	LCP2	5q35.1
576	NM_000433	Hs.587558	NCF2	1q25.3
577	NM_006864	Hs.631592	LILRB3	19q13.42
578	NM_020980	Hs.104624	AQP9	15q22.1
579	NM_002727	Hs.1908	SRGN	10q21.3
580	NM_004049	Hs.227817	BCL2A1	15q25.1
581	NM_002029	Hs.753	FPR1	19q13.33
582	NM_002964	Hs.416073	S100A8	1q21.3
583	NM_002852	Hs.591286	PTX3	3q25.32
584	NM_001001547	Hs.120949	CD36	7q21.11
585	NM_052960	Hs.422688	RBP7	1p36.22
586	NM_005623	Hs.271387	CCL8	17q12
587	NM_181755	Hs.195040	HSD11B1	1q32.2
588	NM_002982	Hs.303649	CCL2	17q12
589	NM_182566	Hs.122561	VMO1	17p13.2
590	NM_004994	Hs.297413	MMP9	20q13.12
591	NM_006770	Hs.67726	MARCO	2q14.2
592	NM_000582	Hs.313	SPP1	4q22.1
593	NM_024508	Hs.136912	ZBED2	3q13.13
594	L34088	Hs.387679	HLA-DQA1	
595	NM_020056	Hs.591798	HLA-DQA2	6p21.32
596	X12544	Hs.654405	X12544	6p21.32
597	NM_002996	Hs.531668	CX3CL1	16q13
598	NM_030643	Hs.115099	APOL4	22q12.3
599	NM_001165	Hs.127799	BIRC3	11q22.2
600	NM_003004	Hs.558009	SECTM1	17q25.3
601	NM_016135	Hs.272398	ETV7	6p21.31
602	NM_005409	Hs.632592	CXCL11	4q21.1
603	NM_006398	Hs.44532	UBD	6p22.1
604	NM_001010919	Hs.381220	FAM26F	6q22.1
605	NM_007315	Hs.642990	STAT1	2q32.2
606	NM_002053	Hs.62661	GBP1	1p22.2
607	NM_002416	Hs.77367	CXCL9	4q21.1
608	NM_001565	Hs.632586	CXCL10	4q21.1
609	NM_052941	Hs.409925	GBP4	1p22.2
610	NM_002164	Hs.840	INDO	8p11.21
611	NM_173574	Hs.353208	ZNF683	1p36.11
612	NM_001768	Hs.85258	CD8A	2p11.2
613	NM_033423	Hs.348264	GZMH	14q12
614	NM_002985	Hs.514821	CCL5	17q12
615	NM_002286	Hs.409523	LAG3	12p13.31
616	NM_006144	Hs.90708	GZMA	5q11.2
617	NM_006433	Hs.105806	GNLY	2p11.2
618	NM_002995	Hs.546295	XCL1	1q24.2
619	NM_006399	Hs.509964	BATF	14q24.3
620	THC2657355		THC2657355	7q22.3
621	NM_002838	Hs.654514	PTPRC	1q31.3

622	NM_001778	Hs.243564	CD48	1q23.3
623	NM_002258	Hs.169824	KLRB1	12p13.31
624	NM_001803	Hs.276770	CD52	1p36.11
625	NM_006564	Hs.34526	CXCR6	3p21.31
626	NM_000732	Hs.504048	CD3D	11q23.3
627	NM_198196	Hs.142023	CD96	3q13.2
628	NM_198517	Hs.534648	TBC1D10C	11q13.1
629	M20432	Hs.409934	HLA-DQB1	6p21.32
630	NM_002122	Hs.387679	HLA-DQA1	6p21.32
631	NM_198232	Hs.78224	RNASE1	14q11.2
632	NR_003491	Hs.517502	MIAT	22q12.1
633	NM_000417	Hs.231367	IL2RA	10p15.1
634	NM_006840	Hs.655338	LILRB5	19q13.42
635	M27126	Hs.654405	M27126	
636	NM_004355	Hs.436568	CD74	5q33.1
637	NM_002118	Hs.654428	HLA-DMB	6p21.32
638	NM_002121	Hs.485130	HLA-DPB1	6p21.32
639	NR_001435		HLA-DPB2	6p21.32
640	BE926212		BE926212	4p13
641	NM_021983	Hs.654405	HLA-DRB4	
642	BC106057	Hs.654405	HLA-DRB3	6p21.32
643	NM_002125	Hs.534322	HLA-DRB5	6p21.32
644	NM_002123	Hs.409934	HLA-DQB1	6p21.32
645	NM_000952	Hs.433540	PTAFR	1p35.3
646	NM_052942	Hs.513726	GBP5	1p22.2
647	NM_001953	Hs.592212	ECGF1	22q13.33
648	NM_033050	Hs.279575	SUCNR1	3q25.1
649	NM_002988	Hs.143961	CCL18	17q12
650	ENST00000368054	Hs.610260	CD84	1q23.2
651	NM_000211	Hs.375957	ITGB2	21q22.3
652	NM_001005340	Hs.190495	GPNMB	7p15.3
653	NM_001645	Hs.110675	APOC1	19q13.32
654	NM_000041	Hs.654439	APOE	19q13.32
655	THC2582296	Hs.654439	THC2582296	19q13.32
656	NM_001017986	Hs.534956	FCGR1B	1p11.2
657	NM_138715	Hs.147635	MSR1	8p22
658	BC031655	Hs.10649	C1orf38	1p35.3
659	NM_007268	Hs.8904	VSIG4	Xq12
660	NM_004244	Hs.504641	CD163	12p13.31
661	NM_024021	Hs.325960	MS4A4A	11q12.2
662	NM_000569	Hs.372679	FCGR3A	1q23.3
663	NM_004172	Hs.481918	SLC1A3	5p13.2
664	BC022548	Hs.125293	RASGEF1A	10q11.21
665	NM_005532	Hs.532634	IFI27	14q32.13
666	NM_004335	Hs.118110	BST2	19p13.11
667	NM_017631	Hs.591710	FLJ20035	4q32.3
668	NM_006417	Hs.82316	IFI44	1p31.1
669	NM_001547	Hs.437609	IFIT2	10q23.31
670	NM_001548	Hs.20315	IFIT1	10q23.31
671	NM_080657	Hs.17518	RSAD2	2p25.2
672	NM_006820	Hs.389724	IFI44L	1p31.1
673	NM_002462	Hs.517307	MX1	21q22.3

674	NM_005101	Hs.458485	ISG15	1p36.33
675	NM_004796	Hs.368307	NRXN3	14q31.1
676	NM_017855	Hs.143811	ODAM	4q13.3
677	AB007954	Hs.604754	KIAA0485	1p31.3
678	NM_017415	Hs.655084	KLHL3	5q31.2
679	BX537788	Hs.688969	BX537788	10p14
680	NM_032679	Hs.148322	ZNF577	19q13.33
681	AK125482	Hs.591496	MEX3A	1q22
682	XM_044166	Hs.591496	MEX3A	1q22
683	NM_005723	Hs.591706	TSPAN5	4q23
684	AK055659	Hs.591706	TSPAN5	4q23
685	BC010538	Hs.657197	C18orf18	18p11.31
686	DQ179139	Hs.558645	C21orf99	9q12
687	NM_003970	Hs.443683	MYOM2	8p23.3
688	NR_003288	Hs.674810	LOC729603	6q25.3
689	AK128234	Hs.416925	CEACAM19	19q13.31
690	BC030123	Hs.163155	LOC441461	9q31.1
691	BC062758	Hs.571424	BC062758	8q21.11
692	BC034319	Hs.363526	BC034319	8p12
693	NM_014289	Hs.496593	CAPN6	Xq22.3
694	NM_005654	Hs.519445	NR2F1	5q15
695	NM_004058	Hs.584744	CAPS	19p13.3
696	AB051549	Hs.525247	ZFHX2	14q11.2
697	NM_030770	Hs.46720	TMPRSS5	11q23.2
698	NM_152529	Hs.516604	GPR155	2q31.1
699	NM_001033045	Hs.516604	GPR155	2q31.1
700	NM_030817	Hs.23388	APOLD1	12p13.1
701	NM_032866	Hs.148989	CGNL1	15q21.3
702	NM_021069	Hs.655143	SORBS2	4q35.1
703	AK094603		AK094603	4q35.1
704	NM_001853	Hs.126248	COL9A3	20q13.33
705	NM_014454	Hs.591336	SESN1	6q21
706	THC2651501		THC2651501	2q14.2
707	NM_002193	Hs.1735	INHBB	2q14.2
708	NM_003483	Hs.505924	HMGA2	12q14.3
709	THC2655811		THC2655811	12q14.3
710	NM_000131	Hs.36989	F7	13q34
711	A_24_P863363		A_24_P863363	14q23.1
712	NM_207381	Hs.306343	TNFAIP8L3	15q21.2
713	NM_001007139	Hs.523414	IGF2	11p15.5
714	NM_001971	Hs.348395	ELA1	12q13.13
715	THC2647658		THC2647658	5q35.3
716	NM_000612	Hs.523414	IGF2	11p15.5
717	THC2644672		THC2644672	16p13.3
718	AK074614		AK074614	11p15.5
719	AL117401	Hs.534980	DKFZP434P211	22q11.23
720	THC2532455		THC2532455	2q37.3
721	BC104421	Hs.645610	BC104421	11p15.5
722	NM_000371	Hs.427202	TTR	18q12.1
723	NM_001063	Hs.518267	TF	3q22.1
724	AK055418	Hs.592518	AK055418	10q21.1
725	NM_006516	Hs.473721	SLC2A1	1p34.2

726	NM_001202	Hs.68879	BMP4	14q22.2
727	NM_004626	Hs.108219	WNT11	11q13.5
728	NM_002899	Hs.529571	RBP1	3q23
729	NR_002196	Hs.533566	H19	11p15.5
730	NM_015432	Hs.188781	PLEKHG4	16q22.1
731	A_24_P916853		A_24_P916853	8q24.21
732	AK097639	Hs.355950	CBWD3	9p11.2
733	NM_198853	Hs.645328	TRIM74	7q11.23
734	A_24_P852099		A_24_P852099	
735	CR627362	Hs.654650	LOC595101	16p11.2
736	THC2551927		THC2551927	3q28
737	AK021738	Hs.632227	TMC6	17q25.3
738	AK057576	Hs.675850	AK057576	12q24.33
739	THC2529512		THC2529512	19q13.11
740	L40520	Hs.654587	L40520	5q13.2
741	AL512711	Hs.26593	HDAC10	22q13.33
742	THC2781073		THC2781073	16q22.1
743	AF289610	Hs.675915	AF289610	2p11.1
744	THC2510656		THC2510656	1q21.1
745	NR_003370	Hs.348979	LOC730092	16p12.1
746	NR_001442	Hs.72222	FER1L4	20q11.22
747	NM_006887	Hs.503093	ZFP36L2	2p21
748	NM_021990	Hs.22785	GABRE	Xq28
749	AK124788		GGTL3	20q11.22
750	ENST00000378052	Hs.490512	ZNF783	7q36.1
751	BC041959	Hs.655573	BC041959	7q11.21
752	BC010934	Hs.693802	ZBTB20	3q13.31
753	AL136621	Hs.644041	ZMYM2	13q12.11
754	ENST00000379606	Hs.507783	ENST00000379606	13q13.3
755	THC2607363		THC2607363	5p13.3
756	THC2669975		THC2669975	7q31.1
757	THC2764893		THC2764893	3q26.31
758	AK024934	Hs.532921	KIAA1641	2q11.2
759	AK057596	Hs.651675	LOC150759	2q11.2
760	BC015370		BC015370	19q13.41
761	THC2746807		THC2746807	11p15.4
762	NR_002802	Hs.648467	TncRNA	11q13.1
763	THC2664215		THC2664215	11p15.5
764	AK025142		DST	6p12.1
765	AA837799	Hs.677704	AA837799	22q12.3
766	BC051317		RBBP6	16p12.1
767	BX090412	Hs.445847	BX090412	15q25.1
768	AL050097	Hs.667735	DKFZP586B0319	3q29
769	THC2611661		THC2611661	11q21
770	DB111455	Hs.660706	DB111455	11q21
771	THC2697162		THC2697162	3q13.31
772	AK129547	Hs.640055	AK129547	6p22.3
773	NM_014226	Hs.104119	RAGE	14q32.31
774	ENST00000360524	Hs.531547	LOC731479	17p11.2
775	ENST00000339425		ENST00000339425	21q11.2
776	BC016022	Hs.679496	BC016022	
777	NM_014141	Hs.655684	CNTNAP2	7q36.1

778	NM_007069	Hs.502775	HRASLS3	11q12.3
779	NM_000153	Hs.513439	GALC	14q31.3
780	NM_006569	Hs.159525	CGREF1	2p23.3
781	NM_000849	Hs.2006	GSTM3	1p13.3
782	NM_014505	Hs.525529	KCNMB4	12q15
783	NM_013332	Hs.433213	HIG2	7q32.1
784	NM_016354	Hs.235782	SLCO4A1	20q13.33
785	BX640887	Hs.400876	LOC388796	20q11.23
786	ENST00000360896	Hs.400876	ENST00000360896	20q11.23
787	NM_004490	Hs.411881	GRB14	2q24.3
788	NM_152559	Hs.647042	WBSCR27	7q11.23
789	NM_001628	Hs.521212	AKR1B1	7q33
790	NM_001150	Hs.1239	ANPEP	15q26.1
791	NM_133637	Hs.191705	DQX1	2p13.1
792	NR_002729	Hs.349110	MSTP9	1p36.13
793	NM_004170	Hs.444915	SLC1A1	9p24.2
794	NM_001935	Hs.368912	DPP4	2q24.2
795	NM_002259	Hs.512576	KLRC1	12p13.2
796	NM_002260	Hs.591157	KLRC2	12p13.2
797	NM_030806	Hs.497159	C1orf21	1q25.3
798	NM_024420	Hs.497200	PLA2G4A	1q31.1
799	NM_002395	Hs.21160	ME1	6q14.2
800	XM_940314	Hs.144372	NLF2	15q22.2
801	NM_004350	Hs.170019	RUNX3	1p36.11
802	AF289562	Hs.592200	AF289562	22q13.1
803	AK125361	Hs.276808	AK125361	22q13.1
804	NM_004062	Hs.513660	CDH16	16q22.1
805	NM_001080395	Hs.514575	AATK	17q25.3
806	NM_001040105	Hs.271819	MUC17	7q22.1
807	NM_006681	Hs.418367	NMU	4q12
808	AK126431	Hs.656660	LOC654433	2q13
809	NM_017577	Hs.24583	GRAMD1C	3q13.31
810	NM_004293	Hs.494163	GDA	9q21.13
811	NM_020826	Hs.436643	SYT13	11p11.2
812	NM_002847	Hs.490789	PTPRN2	7q36.3
813	NM_001003954	Hs.181107	ANXA13	8q24.13
814	NM_004617	Hs.133527	TM4SF4	3q25.1
815	NM_000531	Hs.117050	OTC	Xp11.4
816	NM_176794	Hs.421848	MRPL43	10q24.31
817	NM_004852	Hs.194725	ONECUT2	18q21.31
818	K03200	Hs.662242	K03200	3q29
819	AK027091	Hs.464404	AK027091	
820	BC071773	Hs.626592	BC071773	
821	NM_024786	Hs.659832	ZDHHC11	5p15.33
822	ENST00000377492		ENST00000377492	9q12
823	NM_001089	Hs.26630	ABCA3	16p13.3
824	NM_153350	Hs.513244	FBXL16	16p13.3
825	AY030238	Hs.552649	LQK1	1q32.3
826	NM_006896	Hs.660918	HOXA7	7p15.2
827	NM_003317	Hs.94367	TITF1	14q13.3
828	A_23_P215382		A_23_P215382	
829	NM_152739	Hs.659350	HOXA9	7p15.2

830	NM_000522	Hs.592172	HOXA13	7p15.2
831	A_32_P234853		A_32_P234853	7p15.2
832	NR_002795	Hs.587427	HOXA11S	7p15.2
833	AK093987	Hs.634354	AK093987	7p15.2
834	NM_006547	Hs.648088	IGF2BP3	7p15.3
835	NM_032892	Hs.578544	FRMD5	15q15.3
836	NM_032876	Hs.655832	JUB	14q11.2
837	NM_032034	Hs.105607	SLC4A11	20p13
838	NM_002639	Hs.55279	SERPINB5	18q21.33
839	NM_012101	Hs.504115	TRIM29	11q23.3
840	NM_032405	Hs.208600	TMPRSS3	21q22.3
841	NM_144505	Hs.104570	KLK8	19q13.33
842	NM_144947	Hs.57771	KLK11	19q13.33
843	NM_002776	Hs.275464	KLK10	19q13.33
844	AK095258	Hs.16622	ZNF185	Xq28
845	NM_016584	Hs.98309	IL23A	12q13.2
846	NM_005139	Hs.480042	ANXA3	4q21.21
847	NM_001197	Hs.475055	BIK	22q13.2
848	NM_005555	Hs.654613	KRT6B	12q13.13
849	NM_005554	Hs.433845	KRT6A	12q13.13
850	NM_005980	Hs.2962	S100P	4p16.1
851	NM_003064	Hs.517070	SLPI	20q13.12
852	BC036550	Hs.371980	LOC650392	16p13.3
853	NM_017899	Hs.525709	TESC	12q24.22
854	NM_007231	Hs.522109	SLC6A14	Xq23
855	NR_003038	Hs.292457	SNHG5	6q14.3
856	THC2539634		THC2539634	6q14.3
857	NM_021021	Hs.655236	SNTB1	8q24.12
858	NM_001003845	Hs.368802	SP5	2q31.1
859	NM_012342	Hs.533336	BAMBI	10p11.23
860	NM_003726	Hs.316931	SKAP1	17q21.32
861	NM_002546	Hs.81791	TNFRSF11B	8q24.12
862	AK023647	Hs.43047	AK023647	21q22.3
863	NM_152773	Hs.135997	MGC33212	3q29
864	THC2697511		THC2697511	Xq25
865	NM_003918	Hs.567381	GYG2	Xp22.33
866	NM_004660	Hs.99120	DDX3Y	Yq11.21
867	NM_001039567	Hs.367761	RPS4Y2	Yq11.223
868	NM_001008	Hs.282376	RPS4Y1	Yp11.31
869	XR_018247	Hs.647382	LOC441774	16q23.1
870	NM_198391	Hs.41296	FLRT3	20p12.1
871	NM_145307	Hs.58559	PLEKHK1	10q21.2
872	NM_001666	Hs.3109	ARHGAP4	Xq28
873	NM_032865	Hs.438292	TNS4	17q21.2
874	NM_003302	Hs.534360	TRIP6	7q22.1
875	ENST00000380456	Hs.375142	RGL3	19p13.2
876	BC049823	Hs.380933	RPL22L1	3q26.2
877	NM_000413	Hs.654385	HSD17B1	17q21.31
878	XM_096733	Hs.150840	C14orf72	14q32.31
879	NM_022908	Hs.84753	NT5DC2	3p21.1
880	NM_006163	Hs.75643	NFE2	12q13.13
881	NM_005217	Hs.654448	DEFA3	8p23.1

882	NM_001030060	Hs.567973	SAMD5	6q24.3
883	NM_016569	Hs.129895	TBX3	12q24.21
884	NM_014899	Hs.445030	RHOBTB3	5q15
885	NM_001013680	Hs.441039	LOC401233	6p25.2
886	NM_001001188	Hs.369759	TRPM2	21q22.3
887	NM_024017	Hs.463350	HOXB9	17q21.32
888	THC2783834		THC2783834	17q21.32
889	NM_024016	Hs.514292	HOXB8	17q21.32
890	NM_018952	Hs.98428	HOXB6	17q21.32
891	NM_002146	Hs.654560	HOXB3	17q21.32
892	NM_000148	Hs.69747	FUT1	19q13.33
893	NM_152573	Hs.657750	RASEF	9q21.32
894	NM_024087	Hs.19404	ASB9	Xp22.2
895	NM_001012761	Hs.526902	RGMB	5q21.1
896	NM_022138	Hs.487200	SMOC2	6q27
897	NM_004864	Hs.616962	GDF15	19p13.11
898	NM_203347	Hs.413902	UNQ2541	9q34.3
899	NM_144691	Hs.653110	CAPN12	19q13.2
900	NM_152599	Hs.213603	FLJ35773	17p13.1
901	NM_000676	Hs.167046	ADORA2B	17p12
902	NM_017650	Hs.21816	PPP1R9A	7q21.3
903	NM_017412	Hs.40735	FZD3	8p21.1
904	NM_145168	Hs.87779	HSPC105	16q23.3
905	THC2657737		THC2657737	12p13.2
906	NM_152496	Hs.534562	MANEAL	1p34.3
907	NM_005472	Hs.523899	KCNE3	11q13.4
908	BC041772	Hs.567664	SPNS2	17p13.2
909	ENST00000261275	Hs.383564	KIAA0574	15q13.1
910	NM_153426	Hs.643588	PITX2	4q25
911	AF026246		AF026246	8p23.1
912	NM_014861	Hs.6168	ATP2C2	16q24.1
913	NM_018667	Hs.368421	SMPD3	16q22.1
914	NM_144603	Hs.191762	NOXO1	16p13.3
915	NM_004443	Hs.2913	EPHB3	3q27.1
916	A_32_P177595		A_32_P177595	5q23.3
917	BE714307	Hs.162585	BE714307	5q23.3
918	NM_001004331	Hs.412138	MGC88374	14q23.3
919	AY358366	Hs.642705	KIAA1324	1p13.3
920	A_32_P224040		A_32_P224040	14q32.2
921	NM_004496	Hs.163484	FOXA1	14q21.1
922	NM_001049	Hs.248160	SSTR1	14q21.1
923	NM_000597	Hs.438102	IGFBP2	2q35
924	NM_000282	Hs.80741	PCCA	13q32.3
925	NM_032342	Hs.655738	C9orf125	9q31.1
926	ENST00000374851	Hs.388742	C9orf125	9q31.1
927	NM_002250	Hs.10082	KCNN4	19q13.31
928	NM_001677	Hs.291196	ATP1B1	1q24.2
929	ENST00000382790	Hs.204044	ENST00000382790	18q21.33
930	NM_013314	Hs.665244	BLNK	10q23.33
931	AK126354	Hs.228320	C10orf81	10q25.3
932	NM_024889	Hs.228320	C10orf81	10q25.3
933	NM_032387	Hs.105448	WNK4	17q21.31

934	NM_015225	Hs.262857	KIAA0367	9q21.13
935	NM_021784	Hs.155651	FOXA2	20p11.21
936	NM_020340	Hs.656215	KIAA1244	6q23.3
937	ENST00000330640	Hs.194408	ENST00000330640	6q23.3
938	BG216262	Hs.637431	BG216262	3q21.2
939	AK074886	Hs.434403	LOC389634	12p13.31
940	AB007953		KIAA0484	8p23.1
941	NM_000689	Hs.76392	ALDH1A1	9q21.13
942	NM_000300	Hs.466804	PLA2G2A	1p36.13
943	A_23_P62857		A_23_P62857	
944	A_23_P410456		A_23_P410456	11p15.4
945	NM_203451	Hs.422375	LOC400120	13q13.3
946	CD607715	Hs.255230	CD607715	7q11.21
947	ENST00000325900	Hs.374414	ENST00000325900	Xq26.3
948	AF516696	Hs.656687	CACNA2D3	3p14.3
949	AK125510	Hs.650801	C1orf104	1q22
950	NM_025193	Hs.460618	HSD3B7	16p11.2
951	NM_014337	Hs.438587	PPIL2	22q11.21
952	AK056520	Hs.694296	FLJ31958	10q11.23
953	THC2780599		THC2780599	17q25.1
954	NM_206836	Hs.15250	PECI	6p25.2
955	A_23_P3083		A_23_P3083	14q22.1
956	NM_002563	Hs.654526	P2RY1	3q25.2
957	BC107798	Hs.631558	TNNT1	19q13.42
958	NM_003020	Hs.156540	SCG5	15q13.3
959	THC2585656	Hs.496631	THC2585656	Xq23
960	NM_024829	Hs.131933	FLJ22662	12p13.1
961	NM_206927	Hs.369520	SYTL2	11q14.1
962	NM_182495	Hs.446760	FAM55B	11q23.2
963	NM_152315	Hs.668531	FAM55A	11q23.2
964	NM_017678	Hs.179100	FAM55D	11q23.2
965	AK094950	Hs.375762	AK094950	15q21.1
966	NM_032413	Hs.112242	C15orf48	15q21.1
967	NM_021199	Hs.511251	SQRDL	15q21.1
968	NM_018690	Hs.200333	APOB48R	16p11.2
969	NM_030926	Hs.111577	ITM2C	2q37.1
970	NM_018700	Hs.519514	TRIM36	5q22.3
971	NM_017636	Hs.467101	TRPM4	19q13.33
972	NM_013261	Hs.527078	PPARGC1A	4p15.2
973	ENST00000251847	Hs.101774	ENST00000251847	20p12.1
974	NM_002456	Hs.89603	MUC1	1q22
975	NM_018406	Hs.369646	MUC4	3q29
976	NM_025080	Hs.535326	ASRGL1	11q12.3
977	NM_000240	Hs.183109	MAOA	Xp11.3
978	NM_003645	Hs.11729	SLC27A2	15q21.2
979	NM_000901	Hs.163924	NR3C2	4q31.23
980	NM_015393	Hs.105460	DKFZP564O0823	4q13.3
981	NM_138409	Hs.370055	C6orf117	6q14.3
982	NM_018423	Hs.24979	STYK1	12p13.2
983	NM_017821	Hs.524626	RHBDL2	1p34.3
984	NM_138805	Hs.61265	FAM3D	3p14.2
985	NM_003561	Hs.567366	PLA2G10	16p13.12

986	NM_003196	Hs.446354	TCEA3	1p36.12
987	NM_006633	Hs.291030	IQGAP2	5q13.3
988	NM_153229	Hs.224630	TMEM92	17q21.33
989	NM_016357	Hs.525419	LIMA1	12q13.13
990	NM_001015880	Hs.524491	PAPSS2	10q23.2
991	NM_001218	Hs.210995	CA12	15q22.2
992	NM_001200	Hs.73853	BMP2	20p12.3
993	NM_025047	Hs.287702	ARL14	3q26.1
994	NM_002245	Hs.208544	KCNK1	1q42.2
995	NM_001993	Hs.62192	F3	1p21.3
996	A_23_P103951		A_23_P103951	1q32.2
997	NM_000574	Hs.527653	CD55	1q32.2
998	NM_000716	Hs.99886	C4BPB	1q32.2
999	NM_016619	Hs.546392	PLAC8	4q21.22
1000	NM_025214	Hs.120790	CCDC68	18q21.2
1001	NM_153840	Hs.256897	GPR110	6p12.3
1002	ENST00000267857	Hs.194710	GCNT3	15q22.2
1003	NM_004751	Hs.194710	GCNT3	15q22.2
1004	NM_017723	Hs.657689	C9orf167	9q34.3
1005	ENST00000366874		ENST00000366874	1q41
1006	NM_139248	Hs.68864	LIPH	3q27.2
1007	BM768581	Hs.351126	BM768581	3q27.2
1008	NM_020672	Hs.288998	S100A14	1q21.3
1009	NM_015873	Hs.103665	VILL	3p22.2
1010	NM_181644	Hs.567714	MFSD4	1q32.1
1011	NM_001285	Hs.194659	CLCA1	1p22.3
1012	THC2654007		THC2654007	2p14
1013	NM_152338	Hs.632195	ZG16	
1014	NM_017625	Hs.50813	ITLN1	1q23.3
1015	NM_138938	Hs.567312	REG3A	2p12
1016	NM_001008387	Hs.447084	REG3G	2p12
1017	NM_006507	Hs.4158	REG1B	2p12
1018	NM_002909	Hs.49407	REG1A	2p12
1019	NM_001002236	Hs.525557	SERPINA1	14q32.13
1020	NM_007329	Hs.279611	DMBT1	10q26.13
1021	NM_000439	Hs.78977	PCSK1	5q15
1022	NM_021800	Hs.260720	DNAJC12	10q21.3
1023	NM_001085	Hs.534293	SERPINA3	14q32.13
1024	BX104982	Hs.192124	BX104982	3p22.1
1025	NM_002407	Hs.97644	SCGB2A1	11q12.3
1026	NM_005559	Hs.270364	LAMA1	18p11.31
1027	BC087840	Hs.57971	HES5	1p36.32
1028	AK126207	Hs.299329	B3GNT7	2q37.1
1029	A_32_P169353		A_32_P169353	2q37.1
1030	NM_032717	Hs.99196	MAG1	4q21.23
1031	NM_005209	Hs.415790	CRYBA2	2q35
1032	NM_024533	Hs.156784	CHST5	16q23.1
1033	NM_181726	Hs.693753	ANKRD37	4q35.1
1034	NM_022073	Hs.135507	EGLN3	14q13.1
1035	AK123483	Hs.446388	AK123483	11p15.5
1036	NM_024590	Hs.22895	ARSJ	4q26
1037	NM_004633	Hs.25333	IL1R2	2q11.2

1038	NM_198569	Hs.318894	GPR126	6q24.1
1039	NM_000436	Hs.278277	OXCT1	5p13.1
1040	A_23_P120644		A_23_P120644	20q11.23
1041	NM_014056	Hs.7917	HIGD1A	3p22.1
1042	NM_004235	Hs.376206	KLF4	9q31.2
1043	NM_033342	Hs.487412	TRIM7	5q35.3
1044	NM_138340	Hs.397978	ABHD3	18q11.2
1045	NM_012465	Hs.154296	TLL2	10q24.1
1046	THC2660636		THC2660636	17p13.1
1047	NM_007312	Hs.75619	HYAL1	3p21.31
1048	NM_004970	Hs.839	IGFALS	16p13.3
1049	NM_138969	Hs.170673	RDHE2	8q12.1
1050	NM_005423	Hs.2979	TFF2	21q22.3
1051	NM_002257	Hs.123107	KLK1	19q13.33
1052	NM_174953	Hs.513870	ATP2A3	17p13.2
1053	NM_005476	Hs.5920	GNE	9p13.3
1054	NM_024101	Hs.102406	MLPH	2q37.3
1055	BC022217	Hs.132340	C6orf85	6p25.2
1056	AK091508		AK091508	9q12
1057	NM_139242	Hs.531615	MTFMT	15q22.31
1058	THC2647276		THC2647276	8q24.13
1059	NM_006278	Hs.591947	ST3GAL4	11q24.2
1060	NM_174921	Hs.576320	C4orf34	4p14
1061	A_24_P798431		A_24_P798431	4p14
1062	NM_003225	Hs.162807	TFF1	21q22.3
1063	BC010526	Hs.383197	LOC401127	4p14
1064	THC2736540		THC2736540	21q22.3
1065	NM_006408	Hs.530009	AGR2	7p21.1
1066	NM_176813	Hs.100686	AGR3	7p21.1
1067	NM_018414	Hs.105352	ST6GALNAC1	17q25.1
1068	NM_017417	Hs.511985	GALNT8	12p13.32
1069	NM_182532	Hs.663950	TMEM61	1p32.3
1070	NM_003657	Hs.400556	BCAS1	20q13.2
1071	NM_005187	Hs.513811	CBFA2T3	16q24.3
1072	NM_003890	Hs.111732	FCGBP	19q13.2
1073	NM_032044	Hs.660883	REG4	1p12
1074	ENST00000358378	Hs.534332	ENST00000358378	11p15.5
1075	NR_001558		C20orf56	20p11.21
1076	NM_014471	Hs.555934	SPINK4	9p13.3
1077	NM_198151	Hs.443169	LOC253012	7q21.3
1078	NM_019079	Hs.562195	L1TD1	1p31.3
1079	NM_024565	Hs.14070	CCNJL	5q33.3
1080	NM_032579	Hs.307047	RETNLB	3q13.13
1081	NM_080734	Hs.2719	WFDC2	20q13.12
1082	NM_080736	Hs.2719	WFDC2	20q13.12
1083	NM_033317	Hs.417795	DMKN	19q13.12
1084	NM_024989	Hs.229988	PGAP1	2q33.1
1085	NM_153225	Hs.439040	RPESP	8q21.11
1086	NM_032229	Hs.525105	SLITRK6	13q31.1
1087	NM_004056	Hs.654388	CA8	8q12.1
1088	NM_006533	Hs.646364	MIA	19q13.2
1089	NM_000239	Hs.524579	LYZ	12q15

1090	THC2682885		THC2682885	17q23.1
1091	NM_138799	Hs.467634	MBOAT2	2p25.1
1092	NM_003071	Hs.3068	HLTF	3q24
1093	NM_006072	Hs.131342	CCL26	7q11.23
1094	NM_002888	Hs.131269	RARRES1	3q25.33
1095	NM_020169	Hs.478067	LXN	3q25.32
1096	NM_014751	Hs.336994	MTSS1	8q24.13
1097	NM_002863	Hs.282417	PYGL	14q22.1
1098	NM_014747	Hs.654808	RIMS3	1p34.2
1099	NM_002353	Hs.23582	TACSTD2	1p32.1
1100	NM_001311	Hs.70327	CRIP1	14q32.33
1101	A_23_P21882		A_23_P21882	
1102	A_24_P110601		A_24_P110601	22q11.23
1103	NM_018013	Hs.445244	SOBP	6q21
1104	NM_080685	Hs.436142	PTPN13	4q21.3
1105	NM_000853	Hs.268573	GSTT1	22q11.23
1106	NM_144676	Hs.658619	TMED6	16q22.1
1107	AF275804	Hs.237396	AF275804	16q12.2
1108	BC042557	Hs.31409	LOC120376	11q23.1
1109	NM_030929	Hs.632079	KAZALD1	10q24.31
1110	NM_003695	Hs.415762	LY6D	8q24.3
1111	NM_001505	Hs.20961	GPR30	7p22.3
1112	BE798911	Hs.662555	BE798911	2q31.1
1113	NM_030583	Hs.189445	MATN2	8q22.2
1114	BM802662	Hs.586812	BM802662	13q13.3
1115	NM_000683	Hs.123022	ADRA2C	4p16.2
1116	NM_173660	Hs.122110	DOK7	4p16.2
1117	NM_004004	Hs.591234	GJB2	13q12.11
1118	XM_001131389	Hs.646725	LOC730999	10q22.1
1119	NM_018192	Hs.374191	LEPREL1	3q28
1120	NM_000222	Hs.479754	KIT	4q12
1121	NM_005130	Hs.1690	FGFBP1	4p15.32
1122	NM_001007097	Hs.494312	NTRK2	9q21.33
1123	NM_012385	Hs.513463	NUPR1	16p11.2
1124	NM_000163	Hs.125180	GHR	5p12
1125	NM_020436	Hs.517113	SALL4	20q13.2
1126	BC041856	Hs.536395	DUXAP10	14q11.1
1127	BG118529	Hs.536633	BG118529	14q11.1
1128	NM_005769	Hs.251383	CHST4	16q22.3
1129	NM_153488	Hs.670781	MAGEA2B	Xq28
1130	NM_005367	Hs.169246	MAGEA12	Xq28
1131	NM_006546	Hs.144936	IGF2BP1	17q21.32
1132	NM_153479	Hs.423718	CSAG1	Xq28
1133	NM_015973	Hs.278959	GAL	11q13.2
1134	NM_004086	Hs.21016	COCH	14q12
1135	NM_000363	Hs.644596	TNNI3	19q13.42
1136	DQ249310	Hs.644234	UCA1	19p13.12
1137	NM_002704	Hs.2164	PPBP	4q13.3
1138	NM_002619	Hs.81564	PF4	4q13.3
1139	NM_004131	Hs.1051	GZMB	14q12
1140	NM_001276	Hs.382202	CHI3L1	1q32.1
1141	NM_152737	Hs.111164	RNF182	6p23

1142	NM_012168	Hs.132753	FBXO2	1p36.22
1143	NM_198951	Hs.517033	TGM2	20q11.23
1144	NM_012254	Hs.292177	SLC27A5	19q13.43
1145	NM_147175	Hs.385956	HS6ST2	Xq26.2
1146	NM_031901	Hs.405880	MRPS21	1q21.2
1147	NM_004272	Hs.591761	HOMER1	5q14.1
1148	NM_006404	Hs.647450	PROCR	20q11.22
1149	NM_024693	Hs.22242	ECHDC3	10p14
1150	NM_022350	Hs.591249	LRAP	5q15
1151	BP871540	Hs.515176	BP871540	17p11.2
1152	AL833005	Hs.675929	AL833005	
1153	NM_021158	Hs.516826	TRIB3	20p13
1154	NM_058179	Hs.494261	PSAT1	9q21.2
1155	NM_006623	Hs.487296	PHGDH	1p12
1156	ENST00000357303	Hs.24951	ENST00000357303	2q37.3
1157	BM129308	Hs.653792	BM129308	8p21.2
1158	NM_173567	Hs.201555	ABHD7	1p22.1
1159	NM_032181	Hs.302346	TMEM166	2p12
1160	NM_145051	Hs.211374	RNF183	9q32
1161	BX107298	Hs.502418	BX107298	11p11.2
1162	NM_001147	Hs.583870	ANGPT2	8p23.1
1163	ENST00000309178		ENST00000309178	5q34
1164	NM_002300	Hs.446149	LDHB	12p12.1
1165	NM_005329	Hs.592069	HAS3	16q22.1
1166	NM_033300	Hs.576154	LRP8	1p32.3
1167	NM_144492	Hs.660278	CLDN14	21q22.13
1168	NM_174936	Hs.18844	PCSK9	1p32.3
1169	NM_001216	Hs.63287	CA9	9p13.3
1170	NM_015715	Hs.149623	PLA2G3	22q12.2
1171	BC014113	Hs.663029	LOC254848	4p16.1
1172	NM_001236	Hs.154510	CBR3	21q22.12
1173	NM_152450	Hs.531168	FAM81A	15q22.2
1174	NM_018092	Hs.444046	NETO2	16q12.1
1175	NM_139160	Hs.280990	DEPDC7	11p13
1176	AB051443	Hs.505597	KIAA1656	22q12.2
1177	NM_000662	Hs.591847	NAT1	8p22
1178	NM_152562	Hs.33366	CDCA2	8p21.2
1179	NM_001017420	Hs.99480	ESCO2	8p21.1
1180	NM_018492	Hs.104741	PBK	8p21.1
1181	XR_018387	Hs.646624	LOC647298	13q31.1
1182	BC043212	Hs.120196	LOC402125	3p24.3
1183	NM_145060	Hs.134726	C18orf24	18q21.1
1184	ENST00000375256	Hs.494557	ZNF367	9q22.32
1185	NM_024094	Hs.315167	DCC1	8q24.12
1186	NM_018685	Hs.62180	ANLN	7p14.2
1187	NM_001274	Hs.24529	CHEK1	11q24.2
1188	NM_003318	Hs.169840	TTK	6q14.1
1189	NM_080668	Hs.434886	CDCA5	11q13.1
1190	NM_001254	Hs.405958	CDC6	17q21.2
1191	NM_031966	Hs.23960	CCNB1	5q13.2
1192	NM_022346	Hs.567567	NCAPG	4p15.32
1193	NM_001012271	Hs.514527	BIRC5	17q25.3

1194	NM_001809	Hs.1594	CENPA	2p23.3
1195	NM_018131	Hs.14559	CEP55	10q23.33
1196	NM_001786	Hs.334562	CDC2	10q21.2
1197	NM_002497	Hs.153704	NEK2	1q32.3
1198	NM_145697	Hs.651950	NUF2	1q23.3
1199	NM_032117	Hs.294088	MND1	4q31.3
1200	NM_002358	Hs.591697	MAD2L1	4q27
1201	NM_001067	Hs.156346	TOP2A	17q21.2
1202	A_24_P221285		A_24_P221285	Xp11.3
1203	A_32_P152696		A_32_P152696	9p21.3
1204	BC011243	Hs.642598	ZNF587	19q13.43
1205	NM_181803	Hs.93002	UBE2C	20q13.12
1206	NM_005573	Hs.89497	LMNB1	5q23.2
1207	ENST00000335534	Hs.135094	LOC146909	17q21.31
1208	A_32_P124773		A_32_P124773	3q25.31
1209	BC082758	Hs.644701	BC082758	2q35
1210	NM_030641	Hs.257352	APOL6	22q12.3
1211	THC2598362		THC2598362	16p11.2
1212	NM_000023	Hs.463412	SGCA	17q21.33
1213	NM_015668	Hs.120021	RGS22	8q22.2
1214	NM_015229	Hs.22616	KIAA0664	17p13.3
1215	BC031278	Hs.375797	BC031278	1p21.1
1216	THC2739443	Hs.603732	THC2739443	2q32.1
1217	NM_080621	Hs.27189	SAMD10	20q13.33
1218	NM_006439	Hs.584852	MAB21L2	4q31.3
1219	AB016898	Hs.667982	LOC653483	6q27
1220	NM_019060	Hs.110196	CRCT1	1q21.3
1221	THC2655508		THC2655508	3p21.31
1222	NM_024076	Hs.221873	KCTD15	19q13.11
1223	AJ276555	Hs.152129	AJ276555	12q24.11
1224	A_24_P289854		A_24_P289854	11q22.3
1225	AF264625	Hs.675021	AF264625	10q24.32
1226	A_24_P934306		A_24_P934306	1q32.1
1227	NM_052999	Hs.15159	CMTM1	16q21
1228	AA417913	Hs.87889	AA417913	14q32.13
1229	ENST00000331096		ENST00000331096	12q24.11
1230	NM_005309	Hs.103502	GPT	8q24.3
1231	NM_014507	Hs.349111	MCAT	22q13.2
1232	NM_145659	Hs.528111	IL27	16p11.2
1233	NM_173502	Hs.256632	PRSS36	16p11.2
1234	A_24_P752362		A_24_P752362	8p23.1
1235	ENST00000307662	Hs.654723	SYNPO	5q33.1
1236	ENST00000344415	Hs.677674	LOC400581	17p11.2
1237	XR_018043	Hs.646473	LOC442308	7p11.2
1238	NM_001004313	Hs.462230	LOC388335	17p13.1
1239	NM_000204	Hs.312485	CFI	4q25
1240	ENST00000301171	Hs.250615	ENST00000301171	19q13.2
1241	ENST00000342584	Hs.481464	ENST00000342584	5p15.33
1242	NM_152997	Hs.320147	C4orf7	4q13.3
1243	NM_001192	Hs.2556	TNFRSF17	16p13.13
1244	BC107852	Hs.449585	BC107852	22q11.22
1245	CD709370	Hs.559369	CD709370	22q11.22

1246	ENST00000390256	Hs.570141	ENST00000390256	2p11.2
1247	A_24_P930327		A_24_P930327	19q13.2
1248	ENST00000390559	Hs.510635	ENST00000390559	14q32.33
1249	ENST00000283694	Hs.458446	ENST00000283694	15q11.2
1250	AK026408	Hs.449601	AK026408	22q11.22
1251	ENST00000390625	Hs.631480	ENST00000390625	14q32.33
1252	NM_001995	Hs.406678	ACSL1	4q35.1
1253	BQ880155	Hs.604483	BQ880155	10q11.23
1254	A_23_P72330		A_23_P72330	
1255	BC063599	Hs.449621	IGKV2-24	2p11.2
1256	A_24_P384604		A_24_P384604	2q11.2
1257	ENST00000390260	Hs.449621	ENST00000390260	2p11.2
1258	BC034142	Hs.449621	IGKV1-5	2p11.2
1259	ENST00000390267	Hs.449609	ENST00000390267	2p11.2
1260	ENST00000327926	Hs.525894	ENST00000327926	22q11.1
1261	A_24_P203886		A_24_P203886	2q11.2
1262	ENST00000312946		ENST00000312946	2p11.2
1263	THC2497143	Hs.449585	THC2497143	22q11.22
1264	ENST00000390312	Hs.449585	ENST00000390312	22q11.22
1265	ENST00000390294	Hs.655198	ENST00000390294	22q11.22
1266	BC012876	Hs.449585	BC012876	22q11.22
1267	BC023973	Hs.535668	IGLV6-57	22q11.22
1268	A_23_P159163		A_23_P159163	
1269	A_23_P84791		A_23_P84791	
1270	AY998685	Hs.652878	AY998685	
1271	ENST00000390243	Hs.652878	ENST00000390243	2p11.2
1272	NM_021181	Hs.517265	SLAMF7	1q23.3
1273	AF063695	Hs.449585	AF063695	
1274	A_23_P72252		A_23_P72252	
1275	X57802	Hs.449585	X57802	22q11.22
1276	NM_198440	Hs.593679	DERL3	22q11.23
1277	X57818	Hs.449585	X57818	22q11.22
1278	AF035035	Hs.574701	IGKV1D-8	2p11.2
1279	NM_006235	Hs.654525	POU2AF1	11q23.1
1280	AF267875	Hs.535668	AF267875	22q11.22
1281	X01147	Hs.654512	X01147	22q11.22
1282	A_23_P61068		A_23_P61068	
1283	AJ399872	Hs.449621	AJ399872	
1284	ENST00000390595	Hs.510635	ENST00000390595	14q32.33
1285	AJ252276	Hs.510635	AJ252276	2q11.1
1286	A_23_P113056		A_23_P113056	14q32.33
1287	BC070333	Hs.634941	IGHV1-69	14q32.33
1288	ENST00000390610	Hs.644810	ENST00000390610	14q32.33
1289	ENST00000390622	Hs.644810	ENST00000390622	14q32.33
1290	NM_002281	Hs.658118	KRT81	12q13.13
1291	A_24_P204574		A_24_P204574	22q11.22
1292	A_23_P112957		A_23_P112957	22q11.22
1293	AF076205	Hs.449574	AF076205	22q11.22
1294	BC009479		TTLL3	3p25.3
1295	NM_016459	Hs.409563	MGC29506	5q31.2
1296	BI521983	Hs.449574	ARPC1B	22q11.22
1297	ENST00000328419		ENST00000328419	18p11.31

1298	A_23_P435390		A_23_P435390	
1299	AY172962	Hs.449585	AY172962	22q11.22
1300	NM_001013618	Hs.567636	CTA-246H3.1	22q11.23
1301	NM_020070	Hs.348935	IGLL1	22q11.23
1302	BC024289	Hs.510635	IFI6	14q32.33
1303	ENST00000390543	Hs.510635	ENST00000390543	14q32.33
1304	ENST00000390630	Hs.643624	ENST00000390630	14q32.33
1305	A_23_P158868		A_23_P158868	
1306	AJ245002	Hs.631647	AJ245002	15q11.2
1307	AF067420	Hs.648398	IGHA1	14q32.33
1308	AY003763	Hs.510635	AY003763	
1309	ENST00000390636	Hs.547404	ENST00000390636	14q32.33
1310	A_24_P323298		A_24_P323298	14q32.33
1311	AF103312	Hs.547404	AF103312	14q32.33
1312	A_24_P110487		A_24_P110487	16p11.2
1313	AB063751	Hs.533963	AB063751	14q32.33
1314	BX640624	Hs.648398	IGHA1	14q32.33
1315	AF471475		AF471475	14q32.33
1316	ENST00000390615	Hs.633485	ENST00000390615	14q32.33
1317	Y11328	Hs.650507	Y11328	16p11.2
1318	AF471454	Hs.633485	AF471454	14q32.33
1319	ENST00000360102		ENST00000360102	16p11.2
1320	A_24_P384119		A_24_P384119	16p11.2
1321	ENST00000390632	Hs.644810	ENST00000390632	14q32.33
1322	AJ519285	Hs.551925	AJ519285	14q32.33
1323	EF178107	Hs.644810	EF178107	14q32.33
1324	NP109842		NP109842	16p11.2
1325	ENST00000390624	Hs.644810	ENST00000390624	14q32.33
1326	A_24_P161853		A_24_P161853	16p11.2
1327	ENST00000390609	Hs.510635	ENST00000390609	14q32.33
1328	AK128476	Hs.648398	IGHA1	14q32.33
1329	NM_012390	Hs.651168	SMR3A	4q13.3
1330	THC2699069		THC2699069	17q21.31
1331	BC073764	Hs.449621	IGKC	2p11.2
1332	BC032451	Hs.449621	BC032451	2p11.2
1333	AY062331	Hs.449621	AY062331	2p11.2
1334	BC008026	Hs.676121	MGC16025	2q37.3
1335	BX110985	Hs.143004	BX110985	3p25.3
1336	ENST00000390263	Hs.693601	ENST00000390263	2p11.2
1337	ENST00000390253	Hs.649954	ENST00000390253	2p11.2
1338	ENST00000390261	Hs.693601	ENST00000390261	2p11.2
1339	ENST00000310579		ENST00000310579	9q12
1340	ENST00000331696		ENST00000331696	2p11.1
1341	BC067092	Hs.449621	IGKC	2p11.2
1342	A_24_P490109		A_24_P490109	9q12
1343	BC030813	Hs.449621	BC030813	2p11.2
1344	ENST00000390281	Hs.449621	ENST00000390281	2p11.2
1345	BC095489	Hs.449621	IGKC	2p11.2
1346	ENST00000390275	Hs.390427	ENST00000390275	2p11.2
1347	A_23_P44053		A_23_P44053	2p11.2
1348	ENST00000390258	Hs.693601	ENST00000390258	2p11.2
1349	ENST00000390271		ENST00000390271	2p11.2

1350	A_24_P413286		A_24_P413286	
1351	ENST00000360329	Hs.649259	ENST00000360329	14q32.33
1352	NM_152513	Hs.116419	RP5-821D11.2	22q13.2
1353	BC022362	Hs.449621	BC022362	2p11.2
1354	NM_004944	Hs.476453	DNASE1L3	3p14.3
1355	NM_144646	Hs.643431	IGJ	4q13.3
1356	AW136683	Hs.643431	AW136683	4q13.3
1357	ENST00000390539	Hs.648398	ENST00000390539	14q32.33
1358	AF343666	Hs.648398	AF343666	1q23.1
1359	NM_153259	Hs.591446	MCOLN2	1p22.3
1360	NM_018284	Hs.656774	GBP3	1p22.2
1361	NM_018974	Hs.567508	UNC93A	6q27
1362	THC2520542		THC2520542	5q23.1
1363	NM_001080535	Hs.149219	LINCR	2q11.2
1364	NM_017662	Hs.272225	TRPM6	9q21.13
1365	THC2709754		THC2709754	13q14.11
1366	NM_001353	Hs.460260	AKR1C1	10p15.1
1367	NM_003739	Hs.78183	AKR1C3	10p15.1
1368	BC040210	Hs.460260	AKR1C1	10p15.1
1369	ENST00000377116	Hs.525093	ENST00000377116	13q31.1
1370	NM_033255	Hs.546467	EPSTI1	13q14.11
1371	ENST00000313624	Hs.546467	EPSTI1	13q14.11
1372	NM_020208	Hs.413095	SLC6A20	3p21.31
1373	NM_000253	Hs.195799	MTTP	4q23
1374	NM_080860	Hs.661069	TSGA2	21q22.3
1375	NM_138809	Hs.192586	CMBL	5p15.2
1376	NM_021147	Hs.3041	CCNU	5q11.2
1377	NM_018670	Hs.447531	MESP1	15q26.1
1378	AL832534	Hs.684035	AL832534	2p22.1
1379	A_24_P358606		A_24_P358606	5q32
1380	S73202		S73202	9q34.11
1381	THC2786072		THC2786072	7q31.1
1382	XR_019510	Hs.650952	LOC652810	Yq11.21
1383	NM_002288	Hs.43803	LAIR2	19q13.42
1384	NM_001710	Hs.69771	CFB	6p21.32
1385	NM_006498	Hs.531776	LGALS2	22q13.1
1386	NM_031409	Hs.46468	CCR6	6q27
1387	NM_000625	Hs.434386	NOS2A	17q11.1
1388	NM_002426	Hs.1695	MMP12	11q22.2
1389	NM_033292	Hs.2490	CASP1	11q22.3
1390	NM_016434	Hs.434878	RTEL1	20q13.33
1391	BC004960	Hs.516108	MGC10955	2p13.1
1392	NM_032945	Hs.434878	TNFRSF6B	20q13.33
1393	NM_145252	Hs.105887	LOC124220	16p13.3
1394	NM_001080494	Hs.112949	C1orf34	1p32.3
1395	NM_148672	Hs.656904	CCL28	5p12
1396	NM_002644	Hs.497589	PIGR	1q32.1
1397	THC2591546	Hs.497589	THC2591546	1q32.1
1398	NM_014080	Hs.71377	DUOX2	15q21.1
1399	NM_005564	Hs.204238	LCN2	9q34.11
1400	BC029479	Hs.525513	LOC283587	14q31.3
1401	NM_024780	Hs.115838	TMC5	16p12.3

1402	NM_005764	Hs.431099	PDZK1IP1	1p33
1403	NM_022154	Hs.288034	SLC39A8	4q24
1404	NM_032744	Hs.126409	C6orf105	6p24.1
1405	NM_000715	Hs.1012	C4BPA	1q32.2
1406	NM_001719	Hs.473163	BMP7	20q13.31
1407	NM_002165	Hs.504609	ID1	20q11.21
1408	CN479126	Hs.504609	CN479126	20q11.21
1409	NM_000681	Hs.249159	ADRA2A	10q25.2
1410	NM_006829	Hs.642660	C10orf116	10q23.2
1411	NM_000494	Hs.117938	COL17A1	10q25.1
1412	NM_001003395	Hs.591347	TPD52L1	6q22.31
1413	NM_144696	Hs.658505	C1orf125	1q25.2
1414	NM_183393	Hs.654933	CADPS	3p14.2
1415	BI836406	Hs.130203	BI836406	4q22.1
1416	AK090515	Hs.181297	LOC283663	15q21.3
1417	NM_014859	Hs.499758	KIAA0672	17p12
1418	NM_001823	Hs.173724	CKB	14q32.33
1419	A_24_P538328		A_24_P538328	16q11.2
1420	NM_025163	Hs.518403	PIGZ	3q29
1421	AK023526	Hs.670477	AK023526	4q22.1
1422	NM_015957	Hs.447794	APIP	11p13
1423	A_24_P170203		A_24_P170203	9q31.2
1424	NM_020997	Hs.656214	LEFTY1	1q42.12
1425	NM_080818	Hs.352218	OXGR1	13q32.1
1426	NM_001482	Hs.75335	GATM	15q21.1
1427	THC2687713		THC2687713	4q33
1428	NM_019000	Hs.481704	FAM134B	5p15.1
1429	THC2675062		THC2675062	2q24.3
1430	NM_194298	Hs.499709	SLC16A9	10q21.1
1431	NM_006834	Hs.287714	RAB32	6q24.3
1432	NM_207578	Hs.487325	PRKACB	1p31.1
1433	NM_001010971	Hs.591445	SAMD13	1p31.1
1434	NM_001077	Hs.575083	UGT2B17	4q13.2
1435	NM_001076	Hs.651166	UGT2B15	4q13.2
1436	A_24_P575267		A_24_P575267	4q13.2
1437	NM_001073	Hs.339811	UGT2B11	4q13.2
1438	NM_001075	Hs.201634	UGT2B10	4q13.2
1439	NM_001074	Hs.654424	UGT2B7	4q13.2
1440	NM_053039	Hs.653154	UGT2B28	4q13.2
1441	NM_153699	Hs.553652	GSTA5	6p12.1
1442	BC064586	Hs.410126	LOC145837	15q24.1
1443	NM_004212	Hs.367833	SLC28A2	15q21.1
1444	NM_153486	Hs.380929	LDHD	16q23.1
1445	NM_170601	Hs.10056	SIAE	11q24.2
1446	BC063383	Hs.306721	LOC400573	17p13.1
1447	NM_031264	Hs.165619	MUPCDH	11p15.5
1448	NM_016341	Hs.655033	PLCE1	10q23.33
1449	NM_080489	Hs.657015	SDCBP2	20p13
1450	A_32_P218707		A_32_P218707	2p11.2
1451	NM_002842	Hs.179770	PTPRH	19q13.42
1452	NM_005629	Hs.540696	SLC6A8	Xq28
1453	NM_024307	Hs.289015	GDPD3	16p11.2

1454	BC103878	Hs.288568	MOGAT2	11q13.5
1455	NM_181795	Hs.486354	PKIB	6q22.31
1456	NM_005771	Hs.179608	DHRS9	2q31.1
1457	AK026328	Hs.232604	FLJ22675	11q13.1
1458	NM_033128	Hs.655515	SCIN	7p21.3
1459	NM_000860	Hs.655491	HPGD	4q34.1
1460	NM_019010	Hs.84905	KRT20	17q21.2
1461	NM_007072	Hs.252351	HHLA2	3q13.13
1462	AF088076	Hs.445045	AF088076	1q23.2
1463	NM_015328	Hs.600789	KIAA0828	7q32.1
1464	NM_020299	Hs.116724	AKR1B10	7q33
1465	XR_018726	Hs.646726	LOC340888	10q21.3
1466	NM_197975	Hs.225949	BTNL3	5q35.3
1467	NM_024850	Hs.189109	BTNL8	5q35.3
1468	NM_032321	Hs.389311	MGC13057	2q32.2
1469	NM_002153	Hs.162795	HSD17B2	16q23.3
1470	NM_001041	Hs.429596	SI	3q26.1
1471	NM_000035	Hs.530274	ALDOB	9q31.1
1472	NM_024803	Hs.163079	TUBAL3	10p15.1
1473	NM_198998	Hs.534650	AQP12A	2q37.3
1474	CR749831	Hs.282795	CR749831	10q11.23
1475	NM_175873	Hs.13308	ANKRD43	5q31.1
1476	NM_005588	Hs.179704	MEP1A	6p12.3
1477	NM_012338	Hs.16529	TSPAN12	7q31.31
1478	NM_004063	Hs.591853	CDH17	8q22.1
1479	AB018258	Hs.109358	ATP10B	5q34
1480	NM_004963	Hs.524278	GUCY2C	12p13.1
1481	NM_024791	Hs.374726	PDZD3	11q23.3
1482	NM_000341	Hs.112916	SLC3A1	2p21
1483	NM_005379	Hs.5394	MYO1A	12q13.3
1484	NM_003869	Hs.282975	CES2	16q22.1
1485	NM_145202	Hs.15951	PRAP1	10q26.3
1486	NM_022097	Hs.178589	LOC63928	16p12.1
1487	AK026416	Hs.280892	FLJ22763	3q13.13
1488	NM_018663	Hs.430299	PXMP2	12q24.33
1489	NM_000017	Hs.507076	ACADS	12q24.31
1490	NM_006149	Hs.5302	LGALS4	19q13.2
1491	NM_003226	Hs.82961	TFF3	21q22.3
1492	NM_005971	Hs.301350	FXYD3	19q13.12
1493	NM_016614	Hs.403010	TTRAP	6p22.2
1494	NM_022901	Hs.128071	LRRC19	9p21.2
1495	A_24_P334208		A_24_P334208	2p11.2
1496	NM_019062	Hs.124835	RNF186	1p36.13
1497	NM_005814	Hs.651244	GPA33	1q24.1
1498	NM_001644	Hs.560	APOBEC1	12p13.31
1499	NM_004616	Hs.170563	TSPAN8	12q21.1
1500	NM_001039792	Hs.208081	UNQ338	9p13.3
1501	NM_003944	Hs.632460	SELENBP1	1q21.3
1502	NM_015480	Hs.293917	PVRL3	3q13.13
1503	BC017572	Hs.293917	PVRL3	3q13.13
1504	NM_002591	Hs.1872	PCK1	20q13.31
1505	NM_001815	Hs.11	CEACAM3	19q13.2

1506	NM_004363	Hs.466814	CEACAM5	19q13.2
1507	NM_006890	Hs.74466	CEACAM7	19q13.2
1508	NM_002483	Hs.466814	CEACAM6	19q13.2
1509	BC005008	Hs.466814	CEACAM6	19q13.2
1510	NM_001712	Hs.512682	CEACAM1	19q13.2
1511	ENST00000343933	Hs.113094	CORO2A	9q22.33
1512	NM_000196	Hs.1376	HSD11B2	16q22.1
1513	AL137761	Hs.8379	FAM110C	2p25.3
1514	THC2650074		THC2650074	9q32
1515	NM_014585	Hs.643005	SLC40A1	2q32.2
1516	NM_000765	Hs.111944	CYP3A7	7q22.1
1517	NM_000777	Hs.150276	CYP3A5	7q22.1
1518	NM_017460	Hs.654391	CYP3A4	7q22.1
1519	NM_001979	Hs.212088	EPHX2	8p21.1
1520	BC038245	Hs.335413	BC038245	22q13.2
1521	CR624517	Hs.586321	CR624517	7p21.3
1522	NM_194284	Hs.183617	CLDN23	8p23.1
1523	NM_005218	Hs.32949	DEFB1	8p23.1
1524	NM_004861	Hs.17958	GAL3ST1	22q12.2
1525	NM_003500	Hs.444959	ACOX2	3p14.3
1526	THC2706212		THC2706212	4p16.1
1527	NM_000903	Hs.406515	NQO1	16q22.1
1528	AK131023	Hs.662541	AK131023	17q25.1
1529	THC2770152		THC2770152	20q11.23
1530	NM_007197	Hs.31664	FZD10	12q24.33
1531	NM_002523	Hs.3281	NPTX2	7q22.1
1532	NM_058186	Hs.473877	FAM3B	21q22.3
1533	NM_004683	Hs.77854	RGN	Xp11.3
1534	NM_004010	Hs.495912	DMD	Xp21.2
1535	THC2729899		THC2729899	4q21.22
1536	NM_002986	Hs.54460	CCL11	17q12
1537	NM_014879	Hs.2465	P2RY14	3q25.1
1538	NM_014479	Hs.521459	ADAMDEC1	8p21.2
1539	CR605298	Hs.676511	LOC283904	16q24.1
1540	NM_024768	Hs.134807	CCDC48	3q21.3
1541	XM_001133802	Hs.111902	LOC729464	16q24.1
1542	NM_033380	Hs.369089	COL4A5	Xq22.3
1543	NM_004101	Hs.42502	F2RL2	5q13.3
1544	NM_000029	Hs.19383	AGT	1q42.2
1545	NM_004887	Hs.483444	CXCL14	5q31.1
1546	NM_001463	Hs.128453	FRZB	2q32.1
1547	NM_021229	Hs.201034	NTN4	12q22
1548	AK091132	Hs.120633	AK091132	11q21
1549	NM_005822	Hs.440168	DSCR1L1	6p12.3
1550	ENST00000256861		ITIH5	10p14
1551	NM_015429	Hs.477015	ABI3BP	3q12.2
1552	NM_002736	Hs.433068	PRKAR2B	7q22.3
1553	NM_004657	Hs.26530	SDPR	2q32.3
1554	NM_022469	Hs.98206	GREM2	1q43
1555	NM_002825	Hs.371249	PTN	7q33
1556	THC2609493		THC2609493	7q31.1
1557	NM_001017915	Hs.601911	INPP5D	2q37.1

1558	NM_032432	Hs.233404	ABLIM2	4p16.1
1559	NM_015205	Hs.29189	ATP11A	13q34
1560	NM_021101	Hs.439060	CLDN1	3q28
1561	NM_033260	Hs.591352	FOXQ1	6p25.3
1562	ENST00000309874	Hs.599221	ENST00000309874	15q15.1
1563	BE004814	Hs.647409	BE004814	6p22.3
1564	NM_002763	Hs.585369	PROX1	1q41
1565	NM_004655	Hs.156527	AXIN2	17q24.1
1566	AF078165	Hs.156527	AXIN2	17q24.1
1567	NM_178493	Hs.106137	NOTUM	17q25.3
1568	NM_033119	Hs.592059	NKD1	16q12.1
1569	NM_153000	Hs.293274	APCDD1	18p11.22
1570	THC2652817		THC2652817	16q12.1
1571	NM_020416	Hs.479069	PPP2R2C	4p16.1
1572	NM_018689	Hs.459088	KIAA1199	15q25.1
1573	NM_006393	Hs.5025	NEBL	10p12.31
1574	NR_002307	Hs.381329	MSX2P	17q22
1575	BC005081	Hs.663621	BCAN	1q23.1
1576	BU540282	Hs.600805	BU540282	2p16.1
1577	NM_002334	Hs.4930	LRP4	11p11.2
1578	AF132203	Hs.597496	AF132203	10q24.31
1579	NM_002083	Hs.2704	GPX2	14q23.3
1580	NM_000740	Hs.7138	CHRM3	1q43
1581	NM_001017928	Hs.220594	CCDC58	3q21.1
1582	BC030122	Hs.559426	BC030122	5q14.3
1583	XM_933693	Hs.181156	LOC440731	1q42.2
1584	D89479	Hs.129742	SULT1B1	4q13.3
1585	NM_014465	Hs.129742	SULT1B1	4q13.3
1586	NM_006418	Hs.559736	OLFM4	13q21.1
1587	BX538051	Hs.149540	BX538051	1q25.2
1588	NM_021010	Hs.655233	DEFA5	8p23.1
1589	NM_001926	Hs.711	DEFA6	8p23.1
1590	NM_006017	Hs.614734	PROM1	4p15.32
1591	NM_024677	Hs.590923	NSUN7	4p14
1592	THC2732721		THC2732721	7q35
1593	NM_032501	Hs.529353	ACSS1	20p11.21
1594	AB075864	Hs.370555	KIAA1984	9q34.3
1595	NM_032867	Hs.128196	MICALCL	11p15.3
1596	NM_006671	Hs.104637	SLC1A7	1p32.3
1597	BC004287	Hs.434957	BC004287	8q21.13
1598	NM_007210	Hs.505575	GALNT6	12q13.13
1599	NM_152673	Hs.599259	MUC20	3q29
1600	AK095831	Hs.34447	AK095831	5p15.2
1601	BF803942	Hs.27621	BF803942	5p15.2
1602	NM_000218	Hs.95162	KCNQ1	11p15.4
1603	NM_001185	Hs.546239	AZGP1	7q22.1
1604	NM_000238	Hs.647099	KCNH2	7q36.1
1605	NM_021258	Hs.110915	IL22RA1	1p36.11
1606	NM_004413	Hs.109	DPEP1	16q24.3
1607	AL359055	Hs.547764	AL359055	7p15.3
1608	THC2512199		THC2512199	7p15.3
1609	BC033117	Hs.224879	LOC645249	7q36.3

1610	AW979273	Hs.598388	AW979273	7p15.3
1611	A_32_P25065		A_32_P25065	19p13.11
1612	NM_000245	Hs.132966	MET	7q31.2
1613	NM_032951	Hs.647055	MLXIPL	7q11.23
1614	NM_021977	Hs.567337	SLC22A3	6q25.3
1615	NM_001955	Hs.511899	EDN1	6p24.1
1616	NM_002655	Hs.14968	PLAG1	8q12.1
1617	AK094963	Hs.657374	FLJ37644	17q24.3
1618	NM_001399	Hs.105407	EDA	Xq13.1
1619	BC032913	Hs.97540	LOC389023	2q14.1
1620	NM_153270	Hs.448572	KLHL34	Xp22.12
1621	NM_005396	Hs.423598	PNLIPRP2	10q25.3
1622	NM_004164	Hs.655516	RBP2	3q23
1623	AK130456	Hs.200573	AK130456	6p22.1
1624	NM_022144	Hs.132957	TNMD	Xq22.1
1625	NM_001045	Hs.591192	SLC6A4	17q11.2
1626	THC2669157		THC2669157	10q22.1
1627	NM_006308	Hs.41707	HSPB3	5q11.2
1628	THC2706471		THC2706471	9q13
1629	AW901755		AW901755	5q15
1630	NM_025152	Hs.288981	NUBPL	14q12
1631	NM_001005353	Hs.10862	AK3L1	1p31.3
1632	NM_080759	Hs.129452	DACH1	13q21.33
1633	BC037328	Hs.49329	BC037328	17q24.3
1634	AY358469	Hs.632511	LOC646627	1q44
1635	NM_019018	Hs.591751	FAM105A	5p15.2
1636	AK125648	Hs.155085	AK125648	5p15.2
1637	NM_000880	Hs.591873	IL7	8q21.12
1638	THC2692456		THC2692456	1p32.1
1639	NM_017781	Hs.272795	CYP2W1	7p22.3
1640	NM_032251	Hs.98564	CCDC88	11q13.1
1641	ENST00000305119	Hs.489355	MUC12	7q22.1
1642	A_32_P112263		A_32_P112263	6q13
1643	NM_004923	Hs.145932	MTL5	11q13.2
1644	NM_018291	Hs.444301	FLJ10986	1p32.1
1645	NM_152891	Hs.280658	PRSS33	16p13.3
1646	NM_030615	Hs.150013	KIF25	6q27
1647	AK093351	Hs.297967	FLJ36032	1q21.3
1648	NM_152358	Hs.414175	C19orf41	19q13.33
1649	XR_017750		NANOS3	19p13.12
1650	NM_025243	Hs.221597	SLC19A3	2q36.3
1651	NM_003636	Hs.440497	KCNAB2	1p36.31
1652	NM_001333	Hs.660866	CTSL2	9q22.33
1653	NM_152709	Hs.37636	STOX1	10q21.3
1654	THC2700133		THC2700133	19p12
1655	NM_006644	Hs.36927	HSPH1	13q12.3
1656	ENST00000379426		ENST00000379426	6p24.1
1657	NM_014467	Hs.306339	SRPX2	Xq22.1
1658	NM_017549	Hs.563491	EPDR1	7p14.1
1659	NM_015352	Hs.472409	POFUT1	20q11.21
1660	NM_080672	Hs.473218	PHACTR3	20q13.32
1661	NM_015515	Hs.9029	KRT23	17q21.2

1662	NM_001010853	Hs.652106	ACY1L2	6q15
1663	NM_003279	Hs.182421	TNNC2	20q13.12
1664	NM_002623	Hs.91161	PFDN4	20q13.2
1665	NM_002467	Hs.202453	MYC	8q24.21
1666	NM_144583	Hs.580464	ATP6V1C2	2p25.1
1667	NM_004485	Hs.591531	GNG4	1q42.3
1668	NM_030667	Hs.160871	PTPRO	12p12.3
1669	NM_016653	Hs.444451	ZAK	2q31.1
1670	NM_004289	Hs.404741	NFE2L3	7p15.2
1671	ENST00000211092	Hs.130714	LOC284422	19p13.3
1672	NM_003260	Hs.332173	TLE2	19p13.3
1673	NM_020686	Hs.336768	ABAT	16p13.2
1674	NM_002657	Hs.154104	PLAGL2	20q11.21
1675	BC010544	Hs.660769	BC010544	19p13.3
1676	NM_000142	Hs.1420	FGFR3	4p16.3
1677	ENST00000353250		PIWIL2	8p21.3
1678	NM_152321	Hs.162143	ERP27	12p12.3
1679	NM_003122	Hs.407856	SPINK1	5q32
1680	NM_001008494	Hs.567637	ISX	22q12.3
1681	NM_001445	Hs.519719	FABP6	5q33.3
1682	THC2671575		THC2671575	
1683	NM_003114	Hs.591866	SPAG1	8q22.2
1684	NM_007052	Hs.592227	NOX1	Xq22.1
1685	NM_013955	Hs.592227	NOX1	Xq22.1
1686	NM_013954	Hs.592227	NOX1	Xq22.1
1687	NM_000790	Hs.359698	DDC	7p12.2
1688	NM_021804	Hs.178098	ACE2	Xp22.2
1689	NM_000047	Hs.386975	ARSE	Xp22.33
1690	THC2696614		THC2696614	Xp22.33
1691	NM_003889	Hs.7303	NR1I2	3q13.33
1692	NM_170693	Hs.300863	SGK2	20q13.12
1693	NM_173216	Hs.207459	ST6GAL1	3q27.3
1694	NM_033427	Hs.592285	CTTNBP2	7q31.2
1695	NM_000492	Hs.489786	CFTR	7q31.2
1696	NM_017763	Hs.656319	RNF43	17q22
1697	NM_005170	Hs.152475	ASCL2	11p15.5
1698	NM_014324	Hs.508343	AMACR	5p13.3
1699	NM_030949	Hs.486798	PPP1R14C	6q25.1
1700	NM_025113	Hs.98117	C13orf18	13q14.12
1701	NM_014298	Hs.513484	QPRT	16p11.2
1702	BG114486	Hs.592544	BG114486	16p11.2
1703	NM_003270	Hs.43233	TSPAN6	Xq22.1
1704	NM_006113	Hs.267659	VAV3	1p13.3
1705	NM_144704	Hs.163543	AIFM3	22q11.21
1706	NM_015265	Hs.516617	SATB2	2q33.1
1707	THC2678045		THC2678045	6q27
1708	NM_000845	Hs.449625	GRM8	7q31.33
1709	NM_000933	Hs.472101	PLCB4	20p12.2
1710	NM_005021	Hs.486489	ENPP3	6q23.2
1711	NM_018296	Hs.125139	LRRC36	16q22.1
1712	NM_003212	Hs.385870	TDGF1	3p21.31
1713	NM_014553	Hs.156471	TFCP2L1	2q14.2

1714	L33930	Hs.644105	CD24	Yq11.222
1715	BX413550	Hs.688325	BX413550	Yq11.222
1716	NM_001657	Hs.270833	AREG	4q13.3
1717	NM_001432	Hs.115263	EREG	4q13.3
1718	NM_018407	Hs.492314	LAPTM4B	8q22.1
1719	NM_003878	Hs.78619	GGH	8q12.3
1720	NM_013296	Hs.584901	GPSM2	1p13.3
1721	NM_001338	Hs.693697	CXADR	21q21.1
1722	AL834140	Hs.677892	AL834140	15q11.2
