# Supplementary material for: Colon cancer molecular subtypes identified by expression profiling and associated to stroma, mucinous type and different clinical behavior
Source: BMC Cancer. 2012 Jun 19;12:260. doi: 10.1186/1471-2407-12-260 (PMC3571914; doi:10.1186/1471-2407-12-260)
Supplement: Additional file 4 — Low-stroma-subtype 167 genes predictor. [file 1471-2407-12-260-S4.pdf]

	Parametric p-value	% CV support	Unique id	GB acc
1	< 1e-07	100	Hs.112949	NM_001080494
2	< 1e-07	100	Hs.282795	CR749831
3	< 1e-07	100	Hs.481704	NM_019000
4	< 1e-07	100	Hs.647064	NM_002889
5	< 1e-07	100	Hs.62886	NM_004684
6	< 1e-07	100	Hs.325960	NM_024021
7	< 1e-07	100	Hs.494173	NM_000700
8	< 1e-07	100	Hs.297413	NM_004994
9	< 1e-07	100	Hs.714347	NM_016206
10	< 1e-07	100	Hs.468675	NM_006474
11	< 1e-07	100	Hs.556600	NM_053025
12	< 1e-07	100	Hs.8904	NM_007268
13	< 1e-07	100	Hs.77274	NM_002658
14	< 1e-07	100	Hs.67726	NM_006770
15	< 1e-07	100	Hs.591346	NM_001901
16	< 1e-07	100	Hs.332197	AF200348
17	< 1e-07	100	Hs.203717	BE710245
18	< 1e-07	100	Hs.475353	NM_014583
19	< 1e-07	100	Hs.583348	NM_002192
20	< 1e-07	100	Hs.143250	NM_002160
21	< 1e-07	100	Hs.154654	NM_000104
22	< 1e-07	100	Hs.302085	NM_000961
23	< 1e-07	100	Hs.479119	NM_053044
24	< 1e-07	100	Hs.465929	NM_001299
25	< 1e-07	100	Hs.363396	NM_001014975
26	< 1e-07	100	Hs.122561	NM_182566
27	< 1e-07	100	Hs.592775	AK023391
28	< 1e-07	100	Hs.522891	NM_000609
29	< 1e-07	100	Hs.122927	NM_024692
30	< 1e-07	100	Hs.343522	NM_001001396
31	< 1e-07	100	Hs.434914	NM_152330
32	< 1e-07	100	Hs.500483	NM_001613
33	< 1e-07	100	Hs.313	NM_000582
34	< 1e-07	100	Hs.654439	CA307890
35	< 1e-07	100	Hs.644633	NM_000362
36	< 1e-07	100	Hs.518662	NM_052966
37	< 1e-07	100	Hs.632466	NM_000396
38	< 1e-07	100	Hs.658956	NM_018004
39	< 1e-07	100	Hs.477128	NM_199511
40	< 1e-07	100	Hs.712631	NM_030786
41	< 1e-07	100	Hs.300772	NM_213674
42	< 1e-07	100	Hs.406475	AW276332
43	< 1e-07	100	Hs.134830	AL359062
44	< 1e-07	100	Hs.44385	NM_006873
45	< 1e-07	100	Hs.172928	Z74615
46	< 1e-07	100	Hs.116471	NM_001797
47	< 1e-07	100	Hs.592317	NM_003239
48	< 1e-07	100	Hs.714027	BE835321
49	< 1e-07	100	Hs.522632	NM_003254
50	< 1e-07	100	Hs.264887	NM_023110
51	< 1e-07	100	Hs.198862	NM_001004019
52	< 1e-07	100	Hs.93675	NM_007021
53	< 1e-07	100	Hs.298198	NM_144601
54	< 1e-07	100	Hs.101302	NM_004370
55	< 1e-07	100	Hs.405614	NM_138455
56	< 1e-07	100	Hs.136348	NM_006475
57	< 1e-07	100	Hs.40098	NM_013372
58	< 1e-07	100	Hs.643338	NM_004598
59	< 1e-07	100	Hs.368921	NM_001856
60	< 1e-07	100	Hs.490203	NM_033138
61	< 1e-07	100	Hs.420269	NM_001849

62	< 1e-07	100	Hs.165859	NM_032208
63	< 1e-07	100	Hs.503911	NM_006169
64	< 1e-07	100	Hs.481022	NM_003013
65	< 1e-07	100	Hs.591133	NM_000138
66	< 1e-07	100	Hs.474053	NM_001848
67	< 1e-07	100	Hs.443625	NM_000090
68	< 1e-07	100	Hs.489142	NM_000089
69	< 1e-07	100	Hs.437191	NM_012232
70	< 1e-07	100	Hs.439463	NM_001129
71	< 1e-07	100	Hs.371147	L12350
72	< 1e-07	100	Hs.438993	NM_005504
73	< 1e-07	100	Hs.21509	NM_000168
74	< 1e-07	100	Hs.99528	BM999343
75	< 1e-07	100	Hs.654370	NM_004460
76	< 1e-07	100	Hs.190495	NM_001005340
77	1.00E-07	100	Hs.651244	NM_005814
78	1.00E-07	100	Hs.525607	NM_006291
79	1.00E-07	100	Hs.696554	NM_004791
80	2.00E-07	100	Hs.170131	AK129956
81	2.00E-07	100	Hs.85862	NM_014476
82	3.00E-07	100	Hs.655832	NM_032876
83	4.00E-07	100	Hs.643005	NM_014585
84	4.00E-07	100	Hs.656904	NM_148672
85	4.00E-07	100	Hs.709761	AW268902
86	4.00E-07	100	Hs.514107	D00044
87	5.00E-07	100	Hs.709230	NM_005086
88	7.00E-07	100	Hs.591853	NM_004063
89	8.00E-07	100	Hs.508716	NM_001846
90	1.00E-06	100	Hs.403933	NM_058229
91	1.90E-06	100	Hs.304475	NM_005565
92	2.00E-06	100	Hs.472101	NM_000933
93	2.10E-06	100	Hs.713533	NM_206943
94	2.40E-06	100	Hs.521432	NM_021020
95	2.60E-06	100	Hs.535845	NM_004348
96	2.60E-06	100	Hs.8867	NM_001554
97	2.70E-06	100	Hs.753	NM_002029
98	2.70E-06	100	Hs.526594	AK023854
99	3.40E-06	100	Hs.296049	NM_002404
100	3.50E-06	100	Hs.177841	NM_030762
101	4.80E-06	100	Hs.293917	BC017572
102	5.10E-06	100	Hs.527973	NM_003955
103	5.20E-06	100	Hs.302257	NM_173653
104	5.20E-06	100	Hs.463035	NM_021939
105	7.20E-06	100	Hs.287714	NM_006834
106	7.40E-06	100	Hs.105460	NM_015393
107	7.80E-06	100	Hs.359698	NM_000790
108	7.80E-06	100	Hs.503074	NM_018043
109	9.20E-06	100	Hs.460109	CB240572
110	1.06E-05	100	Hs.705394	NM_006108
111	1.44E-05	100	Hs.81134	NM_173842
112	1.63E-05	100	Hs.374950	NM_005952
113	2.05E-05	100	Hs.143751	NM_005940
114	2.21E-05	100	Hs.156471	NM_014553
115	2.28E-05	100	Hs.288034	NM_022154
116	2.36E-05	100	Hs.647358	X97261
117	2.48E-05	100	Hs.656629	NM_005947
118	2.92E-05	100	Hs.430299	NM_018663
119	3.37E-05	100	Hs.508343	NM_014324
120	3.69E-05	100	Hs.203691	NM_080927
121	3.74E-05	100	Hs.637017	AK055112
122	3.77E-05	100	Hs.348264	NM_033423
123	3.81E-05	100	Hs.303090	NM_005398
124	3.89E-05	100	Hs.162143	NM_152321

125	5.29E-05	100	Hs.9613	NM_139314
126	5.74E-05	100	Hs.705503	NM_001338
127	6.33E-05	100	Hs.353208	NM_173574
128	7.45E-05	100	Hs.1437	NM_000152
129	7.89E-05	100	Hs.301350	NM_005971
130	9.27E-05	100	Hs.517227	NM_021219
131	0.0001079	100	Hs.227817	NM_004049
132	0.0001178	100	Hs.409934	M20432
133	0.0001206	100	Hs.534322	BC106057
134	0.0001329	100	Hs.162795	NM_002153
135	0.0001433	100	Hs.364941	NM_000862
136	0.0001507	100	Hs.504657	NM_016184
137	0.0001545	100	Hs.111944	NM_000765
138	0.0001565	100	Hs.436657	NM_203339
139	0.0001808	100	Hs.302738	NM_000112
140	0.0001842	100	Hs.433391	NM_005950
141	0.0002089	100	Hs.83169	NM_002421
142	0.0002384	100	Hs.109439	NM_033014
143	0.000239	100	Hs.87779	NM_145168
144	0.0002582	100	Hs.473927	AK074662
145	0.0002838	100	Hs.74466	NM_006890
146	0.0002991	100	Hs.657015	NM_080489
147	0.0003184	100	Hs.591866	NM_003114
148	0.0003211	100	Hs.398157	NM_006622
149	0.0003581	100	Hs.1376	NM_000196
150	0.0003893	100	Hs.289015	NM_024307
151	0.0003963	100	Hs.650822	BC040542
152	0.0004023	100	Hs.5302	NM_006149
153	0.0004612	100	Hs.708051	NM_007315
154	0.0004663	100	Hs.657374	AK094963
155	0.0004855	99	Hs.82848	NM_000450
156	0.0005111	99	Hs.171695	NM_004417
157	0.0005185	96	Hs.512682	NM_001024912
158	0.0005374	95	Hs.111577	NM_030926
159	0.0005462	98	Hs.470887	NM_016315
160	0.0005963	92	Hs.530274	NM_000035
161	0.0006131	89	Hs.444046	NM_018092
162	0.0006235	92	Hs.516105	AI659667
163	0.0006654	79	Hs.510078	NM_005627
164	0.0008028	63	Hs.438462	NM_005951
165	0.0009039	48	Hs.437609	NM_001547
166	0.0009256	43	Hs.125139	NM_018296
167	0.0009684	40	Hs.291030	NM_006633
